# The translational oscillation in oocyte and early embryo development

**DOI:** 10.1093/nar/gkad996

**Published:** 2023-11-10

**Authors:** Rajan Iyyappan, Daria Aleshkina, Hao Ming, Michal Dvoran, Kianoush Kakavand, Denisa Jansova, Edgar del Llano, Lenka Gahurova, Alexander W Bruce, Tomas Masek, Martin Pospisek, Filip Horvat, Michal Kubelka, Zongliang Jiang, Andrej Susor

**Affiliations:** Laboratory of Biochemistry and Molecular Biology of Germ Cells, Institute of Animal Physiology and Genetics of the Czech Academy of Sciences, Rumburska 89, 277 21 Libechov, Czech Republic; Laboratory of Biochemistry and Molecular Biology of Germ Cells, Institute of Animal Physiology and Genetics of the Czech Academy of Sciences, Rumburska 89, 277 21 Libechov, Czech Republic; Department of Animal Sciences, University of Florida, Gainesville, FL 32610, USA; Laboratory of Biochemistry and Molecular Biology of Germ Cells, Institute of Animal Physiology and Genetics of the Czech Academy of Sciences, Rumburska 89, 277 21 Libechov, Czech Republic; Laboratory of Biochemistry and Molecular Biology of Germ Cells, Institute of Animal Physiology and Genetics of the Czech Academy of Sciences, Rumburska 89, 277 21 Libechov, Czech Republic; Laboratory of Biochemistry and Molecular Biology of Germ Cells, Institute of Animal Physiology and Genetics of the Czech Academy of Sciences, Rumburska 89, 277 21 Libechov, Czech Republic; Laboratory of Biochemistry and Molecular Biology of Germ Cells, Institute of Animal Physiology and Genetics of the Czech Academy of Sciences, Rumburska 89, 277 21 Libechov, Czech Republic; Laboratory of Biochemistry and Molecular Biology of Germ Cells, Institute of Animal Physiology and Genetics of the Czech Academy of Sciences, Rumburska 89, 277 21 Libechov, Czech Republic; Laboratory of Early Mammalian Developmental Biology, Department of Molecular Biology & Genetics, Faculty of Science, University of South Bohemia in České Budějovice, Branisovšká 31a, České Budějovice, Czech Republic; Laboratory of RNA Biochemistry, Department of Genetics and Microbiology, Faculty of Science, Charles University, Viničná 5, 128 44 Prague 2, Czech Republic; Laboratory of RNA Biochemistry, Department of Genetics and Microbiology, Faculty of Science, Charles University, Viničná 5, 128 44 Prague 2, Czech Republic; Laboratory of Epigenetic Regulations, Institute of Molecular Genetics of the Czech Academy of Sciences, Videnska 1083, 142 20 Prague 4, Czech Republic; Bioinformatics Group, Division of Molecular Biology, Department of Biology, Faculty of Science, University of Zagreb, 10000, Zagreb, Croatia; Laboratory of Biochemistry and Molecular Biology of Germ Cells, Institute of Animal Physiology and Genetics of the Czech Academy of Sciences, Rumburska 89, 277 21 Libechov, Czech Republic; Department of Animal Sciences, University of Florida, Gainesville, FL 32610, USA; Laboratory of Biochemistry and Molecular Biology of Germ Cells, Institute of Animal Physiology and Genetics of the Czech Academy of Sciences, Rumburska 89, 277 21 Libechov, Czech Republic

## Abstract

Translation is critical for development as transcription in the oocyte and early embryo is silenced. To illustrate the translational changes during meiosis and consecutive two mitoses of the oocyte and early embryo, we performed a genome-wide translatome analysis. Acquired data showed significant and uniform activation of key translational initiation and elongation axes specific to M-phases. Although global protein synthesis decreases in M-phases, translation initiation and elongation activity increases in a uniformly fluctuating manner, leading to qualitative changes in translation regulation via the mTOR1/4F/eEF2 axis. Overall, we have uncovered a highly dynamic and oscillatory pattern of translational reprogramming that contributes to the translational regulation of specific mRNAs with different modes of polysomal occupancy/translation that are important for oocyte and embryo developmental competence. Our results provide new insights into the regulation of gene expression during oocyte meiosis as well as the first two embryonic mitoses and show how temporal translation can be optimized. This study is the first step towards a comprehensive analysis of the molecular mechanisms that not only control translation during early development, but also regulate translation-related networks employed in the oocyte-to-embryo transition and embryonic genome activation.

## Graphical Abstract

**Graphical Abstract:**
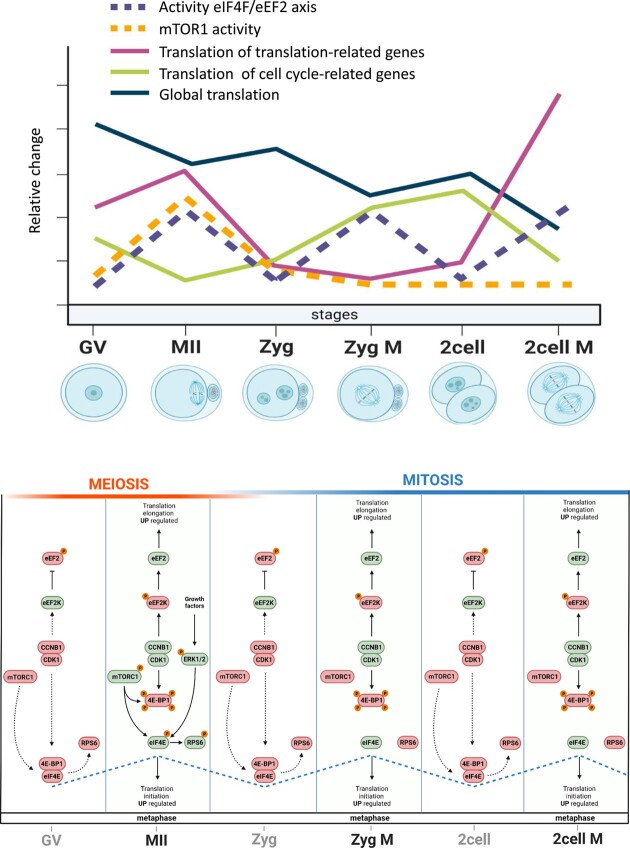

## Introduction

The regulation of gene expression and protein synthesis is a complex and dynamic process that involves translational control, modulating the translation of mRNA into protein. Although mRNA levels are an important measure of gene expression, they may not always correspond directly to protein levels due to various molecular mechanisms that can influence translation. Thus, translational control plays a critical role in maintaining a dynamic system of gene expression and protein synthesis in cells (1). Protein synthesis is especially important for mature mammalian oocytes, that rely solely on pre-synthesised maternal mRNAs, translation and its regulation (2,3). Once the oocyte reaches its fully grown state, referred to as the ‘germinal vesicle stage’ (GV-stage, GV), meiosis is halted during prophase I and transcription is silenced (4). After two asymmetric meiotic divisions with two polar body extrusions (MI and MII) and fertilization, the zygote forms male and female interphase pronuclei. As the pronuclei come together during syngamy, a metaphase plate is established, which triggers the first mitotic division (5). In contrast to non-mammalian vertebrates, zygotic genome activation in mouse occurs at the two-cell stage, however, the first mitotic cleavage is completed relatively late (24 h after fertilization) (6). Once meiosis is resumed, degradation of maternal mRNAs begins and firmly continues until the major genome activation at the two-cell stage (7).

Many mRNAs in the GV oocyte are stored within ribonucleoproteins (RNPs) to prevent their degradation (8). Selective polyadenylation and de-capping are major controlling mechanisms leading to translation regulation, storage or degradation (9,10). After nuclear envelope breakdown (NEBD), the oocyte relies mainly on cap-dependent translation, however, global protein synthesis is downregulated (11). Cap-dependent translation is regulated by binding of the translation repressor eIF4E binding protein 1 (4E-BP1) to the eukaryotic translation initiation factor 4E (eIF4E), preventing translation initiation (12). In addition, translation is controlled by the eukaryotic elongation factor 2 kinase (eEF2K), which phosphorylates and inhibits eEF2 (T56), slowing down the translation elongation step (13). The mammalian oocyte is a large cell and therefore has to control its translation spatially, e.g. several active chromosomal translation hotspots are controlled by mammalian target of rapamycin (mTOR)–eIF4F activity (the mTOR/4F axis) (14).

It is generally accepted that global translation is less active during M-phase in comparison to interphase as a result of translation initiation factor phosphorylation states (reviewed in 15). Despite a significant reduction of translation in both in mitosis (16) and meiosis (17), translation of a subset of mRNAs is upregulated during the M-phase progression, compared to interphase, via upregulation of the mTOR/4F axis (14,18,19). In somatic cells, terminal oligopyrimidine tract (TOP) containing mRNAs are actively translated in mitotic M-phase (20). In mammalian oocytes, there is a unique opportunity to compare the expression of various proteins in the meiotic M-phase or early embryo mitoses and identify specific actively translated mRNAs. The roles of such upregulated mRNAs during M-phase are not well understood and studying their expression in oocytes and early embryos may provide valuable insights into their functions.

Here, we present the patterns of translational regulation in oocyte and early embryo development, with emphasis on the cell cycle. We show highly dynamic quantitative and qualitative changes of translatomes in interphases and M-phases, related to the regulation of cell physiology that orchestrates developmental processes. In addition, our results reveal several candidate genes that may be important for meiotic maturation and early embryonic development.

## Materials and methods

### Oocyte and embryo isolation and cultivation

Oocytes were acquired from ICR mice of a minimum of 6 weeks old. The females were stimulated 46 h prior to oocyte isolation using 5 IU of pregnant mare serum gonadotropin (PMSG; Folligon; Merck Animal Health) per mouse. Fully grown GV oocytes were isolated into transfer medium (TM) supplemented with 100 μM 3-isobutyl-1-methylxanthine (IBMX, Sigma Aldrich) to prevent spontaneous meiotic resumption. Selected oocytes were denuded and cultivated in M16 medium (Merck Millipore) without IBMX at 37°C, 5% CO_2_ for 0 (GV) or 12 h (MII). For embryo collection, the PMSG stimulated mice were injected with 5 IU hCG before being mated overnight with males of the same strain. After 16 h, zygotes were recovered from the excised oviducts and cultured in KSOM medium (Merck Millipore) until two-cell stage. Interphase pronuclei zygotes were collected at the time point of isolation; metaphase zygotes and two-cell embryos were treated with nocodazole (10 uM; M1404, Sigma Aldrich). Embryos were treated with 0.5 mM sodium arsenite in KSOM medium (21). The expected developmental results of the treated cells were normalized to the controls, which were set at 100%. All animal experiments were performed in accordance to guidelines and protocols approved by Laboratory of Biochemistry and Molecular Biology of Germ Cells at the Institute of Animal Physiology and Genetics in Czech Republic (22). All animal work was conducted according to Act No. 246/1992 on the protection of animals against cruelty, issued by experimental project #67756/2020MZE-18134, issued by the Ministry of Agriculture.

### *In vitro* fertilization (IVF)

4-Week old female ICR mice were injected with 5IU of PMSG (ProSpec) 46 h prior to hCG (ProSpec) administration (12–12-day/night cycle). For *in vivo* MII collection, mice were sacrificed 14 h post-hCG injection. Cumulus-oocyte complexes for *in vitro* fertilization (IVF) were collected from ampulla into preheated and equilibrated KSOM medium (Merck). In *vitro* matured denuded MII oocytes were subjected to IVF following 15 h culture in a MEM medium (M0200, Sigma Aldrich). Sperm were retrieved from cauda epididymis of 12-week old males and capacitated for a 1 hour in HTF medium (Merck) supplemented with BSA (BioXtra, Sigma Aldrich) and GSH (BioXtra, Sigma Aldrich). The IVF itself was performed for 4 h in the same media as sperm capacitation followed by switching into KSOM media.

### Inhibitor treatment

Oocytes were treated with selective p70 ribosomal S6 kinase (S6K1) inhibitor p70KI (5 μM; PF-4708671, Selleckchem) or Exotoxin A (72 nM, ETA; P0184, Merck) from 0 (GV) to 16 h (MII) in M16 medium. Zygotes were treated with p70KI or ETA, 0 (Zygote) 20 h (two-cell) then washed and cultured until the blastocyst stage in M16 medium under mineral oil. IVF embryos were treated with p70KI prior to fertilization during oocyte progression from 0 (GV) to 16 h (MII) in M16 medium.

### RNA isolation and qPCR

TRIzol reagent (Invitrogen) was used for RNA extraction following the manufacturer's instructions. Reverse transcription was performed with qPCRBIO cDNA Synthesis Kit (PCR Biosystems). qPCR was then carried out using the QuantStudio 3 (Applied Biosystems) and the Luna® Universal qPCR Master Mix (New England BioLabs) according to manufacturer's protocols with an annealing temperature of 60°C. Primers are listed in Supplementary Table S1A.

### Immunoblotting

An exact number of cells (15–30 oocytes) were washed in PVA/PBS and frozen to − 80°C. Prepared samples were lysed in NuPAGE LDS Sample Buffer (NP0007, Thermo Fisher Scientific) and NuPAGE Sample Reducing Agent (NP0004, Thermo Fisher Scientific) and heated at 100°C for 5 min. Proteins were separated on precast gradient 4–12% SDS–PAGE gel (Thermo Fisher Scientific) and blotted to Immobilon P membrane (Millipore) in a semidry blotting system (Biometra GmbH) at 5 mA cm^2^ for 25 min. Membranes were then blocked in 5% skimmed milk dissolved in 0.05% Tween-Tris buffer saline (TTBS), pH 7.4 for 1 h. Membranes were incubated overnight at 4°C with relevant primary antibodies (Supplementary Table S1B) diluted in 1% milk/TTBS. Appropriate Peroxidase conjugated secondary antibodies were used (711-035-152 Anti-Rabbit Donkey, or 715-035-151 Anti-Mouse Donkey, both Jackson Immunoresearch) at a 1:7500 dilution in 1% milk/TTBS for 1 h at room temperature. ECL (Amersham) was used for visualization of immunodetected proteins on X-ray films. The films were scanned by calibrated densitometer (GS- 800, Bio-Rad Laboratories) and quantified in ImageJ. Presented images were cropped from membranes, contrast and brightness was adjusted using Adobe Photoshop CS3.

### Immunocytochemistry

Fixed oocytes (15 min in 4% PFA, Sigma Aldrich) were permeabilized in 0.1% Triton X-100 for 10 min, washed in PBS supplemented with polyvinyl alcohol (PVA, Sigma Aldrich) and incubated with primary antibodies (Supplementary Table S1B), diluted in PVA/PBS and incubated overnight at 4°C. Oocytes were then washed 2 × 15 min in PVA/PBS and antigen-associated primary antibodies were detected using relevant Alexa Fluor 488/594/647 conjugates (Invitrogen), diluted to 1:250 for 1 h at room temperature. One drop of ActinGreen 488 ReadyProbes Reagent (R37110, Invitrogen) per 10 minute was then used for labelling filamentous actin in each sample (20–30 oocytes per group). Washed oocytes (2 × 15 min in PVA/PBS) were mounted onto slides using ProLong Mounting Medium with DAPI. An inverted confocal microscope (Leica SP5) was used for sample visualization. Image quantification and assembly were performed using ImageJ and Adobe Photoshop CS3. Experiments were repeated three time, with 20–30 oocytes per group/experiment.

### Measurement of overall protein synthesis

To measure the overall protein synthesis, 50 mCi of ^35^S-methionine (Perkin Elmer) was added to methionine-free culture medium. Exact number of oocytes per sample (5–10) were labelled for 12 h, then lysed in SDS-buffer and subjected to SDS–polyacrylamide gel electrophoresis (PAGE). The labelled proteins were visualized by autoradiography on a BasReader (Fuji) and quantified by Aida software (RayTest). GAPDH (G9545, Sigma Aldrich) was used as a loading control.

### *In situ* proximity ligation assay (PLA)

Proximity ligation assays were performed according to manual instructions of the PLA Duolink kit (Sigma Aldrich). Oocytes and embryos were fixed for 15 min in 4% paraformaldehyde in PBS and permeabilized for 10 min in 0.1% Triton X-100 in PBS; PLA Duolink kit blocking solution was added to each sample. Oocytes were incubated with primary antibodies; rabbit anti-RPL24 (PA562450, Thermo Fisher) and mouse anti-RPS6 (74459, Santa Cruz) at 4°C overnight. The samples were washed in PBS and then in Wash Buffer A (Sigma Aldrich). The samples were incubated with 40 μl reaction mixtures (8 μl PLA probe MINUS stock, 8 μl PLA probe PLUS stock and 24 μl PBS) in a chamber for 1 h at 37°C. The slides were then washed in 1x Wash Buffer A for 6 × 2 min and ligation was performed in 40 μl reaction: 1 μl of ligase to 39 μl of ligation solution. Samples were incubated in ligation reaction mixture for 30 min at 37°C then washed 6 × 2 min in Wash Buffer A. 40 μl of amplification reaction (0.5 μl polymerase and 39.5 μl amplification solution) was added to each sample before incubation at 37°C for 100 min. Next, the samples were washed in Wash Buffer B (Sigma Aldrich) for 3 × 5 min and in 0.01% Wash Buffer B for 2 min. The samples were mounted in Vectashield Mounting Medium containing DAPI (Vector Laboratories). Quantification of interaction foci was performed using ImageJ/FIJI. Three experiments with 70 oocytes/embryos each were performed.

### SSP-profiling

Polysome fractionation followed by RNA isolation was carried out according to Scarce Sample Polysome profiling (SSP-profiling) method by (23). Briefly, cycloheximide - treated oocytes (CHX, Sigma Aldrich) were lysed and resulting samples were loaded onto 10–50% sucrose gradients. Centrifugation was performed in the SW55Ti rotor (Beckman Coulter) at 45 000 RPM (246 078 × g) for 65 min at 4°C (Optima L-90 ultracentrifuge, Beckman Coulter). Ten equal fractions were collected from each polysome profile and subjected to RNA isolation by Trizol reagent (Sigma Aldrich). qRT-PCR-based (QuantStudio 3, Applied Biosystems) quantification of 18S and 28S rRNAs in each fraction was applied to visualize individual polysome profiles (23). Sequencing libraries were prepared using SMART-seq v4 ultra low input RNA kit (Takara Bio). Sequencing was performed with Novaseq 6000 (Illumina) as 150-bp paired-end reads. Reads were trimmed using Trim Galore v0.4.1 and mapped onto the mouse GRCm38 genome assembly using STAR (2.5.3a) with default parameters. Individual mapped reads were quantified as fragments per kilobase of exon model per million mapped fragments (FPKM) values with RefSeq genes as reference. Differential expression analysis was performed by a Partek Flow GSA algorithm with default parameters. The genes were deemed differentially expressed if they provided a false discovery rate of < 0.05 and fold change >2. Webgestalt (https://www.webgestalt.org/) was used to reveal the Gene Ontology (GO).

### Statistical analysis and data visualization

For the statistical analysis and data visualization (column charts), GraphPad Prism 8.3 was used. Statistical analysis included Student's *t* tests to determine if the difference between the groups is statistically significant. All experiments were repeated at least three times. The analysis and visual representation of RNA-seq data were done via R studio and PrismaGraph9 software (volcano plots, heatmaps, Vienna diagrams, dot-plots, PCA; box-plots).

## Results

### Global translation is decreased in the M-phase of meiosis and the two subsequent mitoses

**Figure 1.**
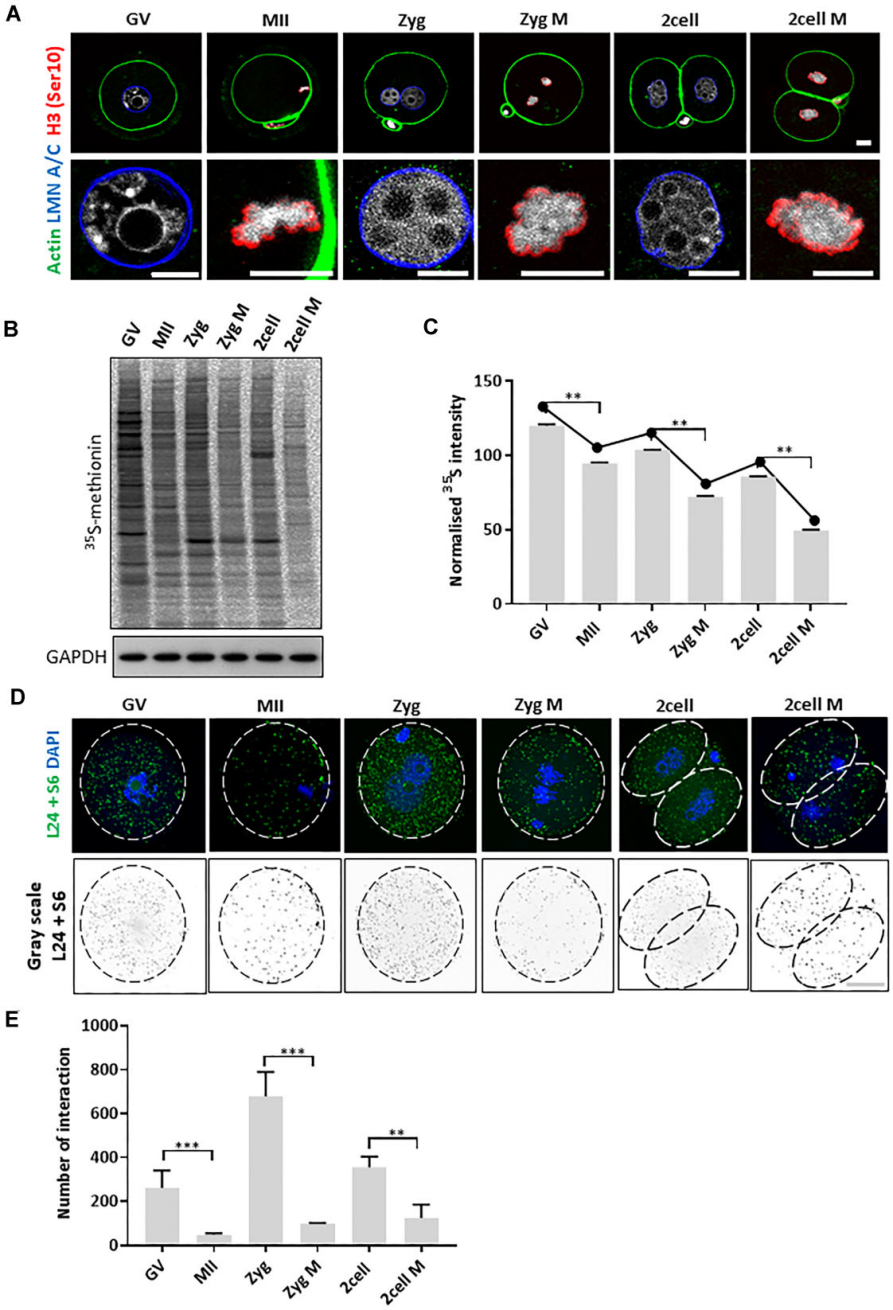
Global translation is decreased in the M-phase of meiosis and the two subsequent mitoses. (**A**) Immunocytochemical analysis of oocytes and early embryos in the interphase (LMN A/C, blue) and M-phase (H3-Ser10, red), DNA labelled with DAPI (gray), Actin (green, cortex). Scale bar, 15 μm. The lower row shows the zoomed nuclei/chromosomal area. (**B**) ^35^S-methionine labelling of oocytes and embryos to visualize global translation of the specific stage of the developing oocyte and early embryo. GAPDH was used as a loading control, *n* ≥ 3. (**C**) Normalized densitometric values of ^35^S-methionine from stages in (B). Data are represented as the mean ± s.d.; **P< 0.01 according to Student's *t*-test, *n* ≥ 3. (**D**) Proximity ligation assay detecting *in situ* ribosome assembly using RPL24 and RPS6 markers (L24 + S6, green and grey dots). The white and black dashed line indicates cellular cortex; representative images from three independent experiments shown. Scale bar, 20 μm. (**E**) Quantification of ribosome assembly in the specific developmental stages. Data are represented as the mean ± s.d.; **P< 0.01 and ***P< 0.001 according to Student's *t*-test; from three independent experiments, *n* ≥ 70. For additional analysis see Supplementary Figure 1.

Due to transcriptional silencing in oocytes and early embryos, mRNA translation is the dominant regulator of oocyte and preimplantation embryo development. To better understand active translation during oocyte (GV and MII) and early embryo development (Zygote and two-cell) we performed a systematic analysis and compared active translation, with emphasis on two major cell cycle phases, interphase and metaphase (M). Timing and sample collection were standardized based on the morphology and immunostaining with an antibody against a nuclear marker (Lamin A/C) and metaphase marker (Histone H3 phosphorylated at Ser130) (Figure 1A), plus the chromosomal and nuclear morphology (Supplementary Figure 1A). Firstly, we performed a ^35^S-Methionine incorporation assay to analyse global translation in GV (meiotic prophase) and MII (meiotic M-phase) oocytes, as well as early embryos in the zygote (Zyg), zygote in the first mitotic M-phase (Zyg M), two-cell (two-cell) and two-cell in the second mitotic M-phase (two-cell M) stages. Consistent with previously published results (15,17), we observed a global decrease in protein synthesis during oocyte and embryo development with a significant decrease during M-phases (Figure 1B, C). To further confirm this finding, we performed proximity ligation assays (PLA) using RPL24 and RPS6 ribosome assembly markers (3). Similarly, ribosome assembly showed decreases from oocyte to embryo stages, with subsequent increases in the zygote and the two-cell stage, yet with concomitant and significant decreases at all M-phases examined (Figure 1D, E). To exclude the possibility that our experimental approach influenced translation, we analysed the activity of the stress marker eIF2α (Ser51) (24), that showed no increase in our samples (Supplementary Figure 1B, C), despite our observation that global translation was significantly decreased (Figure 1B–E). Additionally, we analysed the effect of our synchronisation protocol using nocodazole in the naturally occurring oocyte M-phase. We found no effect of nocodazole on the activity of the stress marker eIF2α (Ser51), phosphorylation of translation initiation and elongation regulators; 4E-BP1 and eEF2K, respectively (Supplementary Figure 1D), suggesting that such treatment does not induce a translational stress response. Additionally, we analyzed phosphorylation of 4E-BP1, eEF2 and eIF2a in the absence or presence of a cellular stressor (sodium arsenite, 22) which shows clear influence in the M-phases on the translational players via dephosphorylation of 4E-BP1 and phosphorylation of eEF2 and eIF2a (Supplementary Figure 1E). Correspondingly, the Gene Ontology (GO) profiles do not show processes related to the stress response or apoptosis (Figure 5F). The results indicate similar trends for active translation at the global scale with significant decrease in the M-phases (Figure 1B–E). Overall, these results suggest that significant translational changes occur in the oocyte and early embryo depending on the cell cycle stage.

### Dynamics of polysome bound mRNAs coding for components of specific biological processes in the oocyte and early embryo development

To decipher the pattern of protein synthesis and its regulation, we conducted Scarce Sample Polysome Profiling (SSP-profiling) to analyse active translation of mRNAs at the genome-wide level (23). An improved SSP-profiling protocol was followed by RNA sequencing (RNA-seq), which allowed us to analyse mRNA translational profiles of mouse oocytes at the GV and MII stages, as well as Zyg, Zyg M, two-cell and two-cell M stages. A total of 10 fractions were separated by polysomal fractionation, from which the first five fractions were pooled and labelled as non-polysome (NP) fractions and the heavier five fractions were pooled and labelled as polysome (P) associated fractions. qRT-PCR analysis quantification of 18S and 28S rRNA (the amount of 18S and 28S rRNA provided an assessment of the reproducibility of collected fractions, Supplementary Figure 2) content confirmed the successful separation of polysome occupied RNA (23). Additionally, principal component analysis (PCA) and clustering analysis of polysome and non-polysome RNA-seq profiles demonstrated the reproducibility of sample preparation and RNA-seq profiles between biological replicates in each group and across the assayed developmental stages (Supplementary Figure 3A–E).

**Figure 2.**
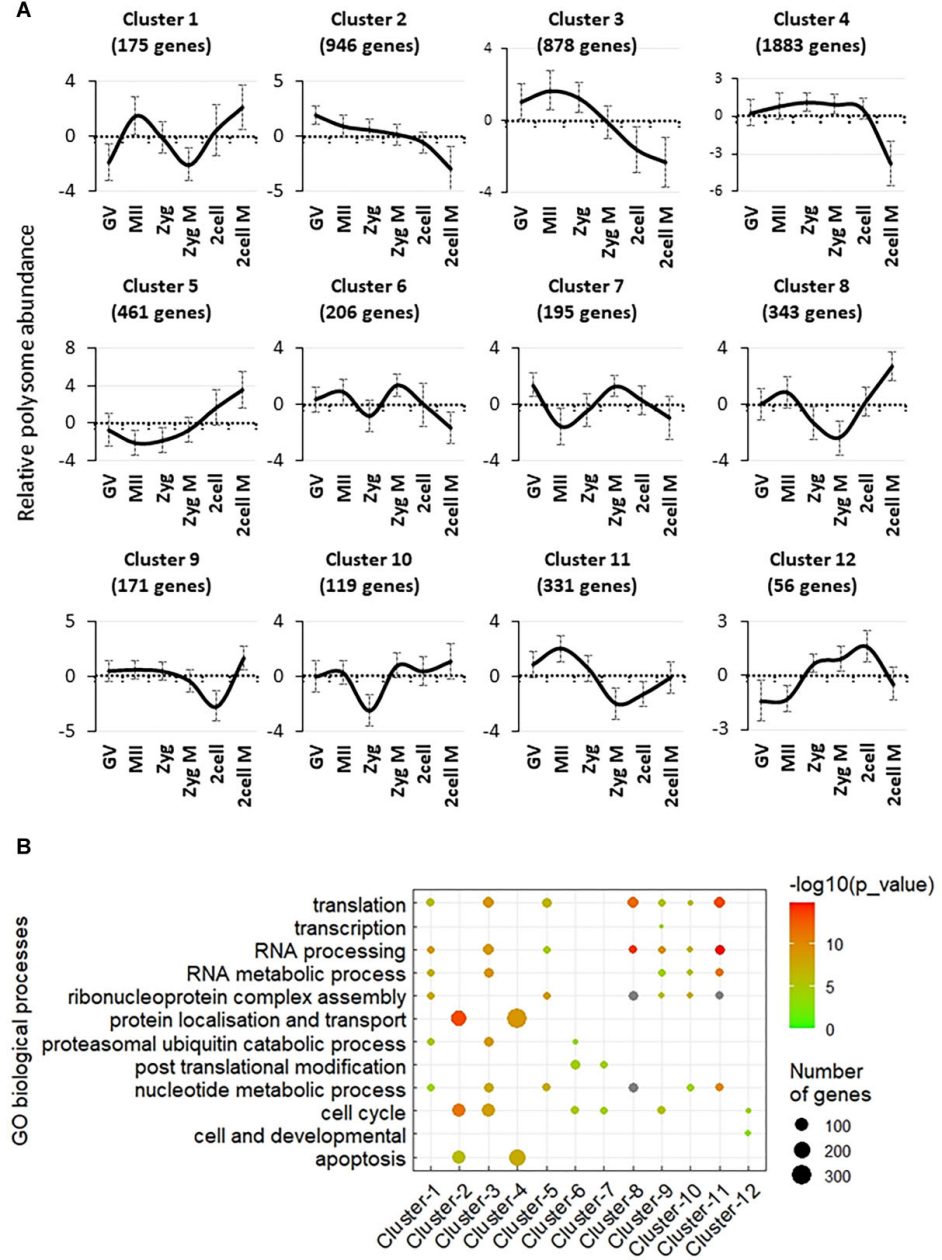
Dynamics of polysome bound mRNAs coding for components of specific biological processes in the oocyte and early embryo development. (**A**) 12 different clusters demonstrate temporal patterns of polysome bound RNAs in the developing oocyte and early embryo. Connected to Supplementary File S1 and Supplementary File 2. (**B**) Gene Ontology categories that relate to distinct clusters (A) are plotted from over representation analysis (WebGestalt). Connected to Supplementary File 3.

We next sought to investigate the regulation of global translation during various stages of oocyte and early embryo development, with a particular emphasis on interphases, meiosis, and the first two embryonic mitoses. First, we characterized the behavior of actively translating mRNAs by analysing the patterns of translational changes between the different stages. A total of 12 distinct clusters exhibiting a specific pattern of mRNAs associated with polysomes were uncovered to statistical significance (*P* < 0.05) (Figure 2A and Supplementary File 2; where genes of each cluster are listed) across all analysed stages. The results clearly show that the translation of maternal mRNAs is highly dynamic and falls into different subgroups representing mRNAs that are important for particular stages. For example, genes falling under clusters 1 and 8 are essential for meiosis and the second mitosis of the embryo. Genes in cluster 10 are mostly expressed in M-phases of meiosis and the first and second embryonic mitoses. Cluster 6 genes are actively translated during the second meiosis of oocyte and the first mitosis in the zygote. Cluster 7 shows a strong association with polysomes only in the first embryonic mitosis. Conversely, cluster 11 is important only for the second meiotic division without a role in earlier development. Translation of mRNAs from clusters 5, 9 and 12 are involved in post-fertilization processes and are necessary for the second embryonic meiosis.

Next, we performed Gene Ontology analysis (GO; Figure 2B) to understand the biological function of each gene cluster. Overall, GO analysis showed that most of the polysome occupied mRNAs belong to biological processes linked to translation, RNA metabolism, proteasome, post-translational modification, apoptosis and cell cycle (Figure 2B and Supplementary File 3).

### Differential perturbations of the translatome depending on developmental and cell cycle stage

We next performed comparative analyses of differential mRNA translation based on developmental stage and cell cycle in connection to Figure 1B–E. For validation we selected candidate genes coding for a key meiotic and mitotic cell cycle factor *Cdc20* (25), Oocyte- and Embryo-Specific Protein 19 (OOEP), 60S Ribosomal Protein L35 (RPL35), MOS Proto-Oncogene, Serine/Threonine Kinase (Mos) and RNA Polymerase II Subunit I (POLR2I) (Supplementary Figure 4). Total mRNA coding for CDC20 is equally expressed in oocytes and embryos except for two-cell stage (two-fold change between two-cell M versus two-cell) (Supplementary Figure 4), however, in the polysomal fractions its mRNA is significantly elevated during the M-phases (Supplementary Figure 4), positively correlating with the CDC20 protein expression profile (Supplementary Figure 4). Similarly, the additional candidate mRNAs showed similar polysomal occupancy measured by qPCR (Supplementary Figure 4) and positively correlated with protein expression (Supplementary Figure 4).

**Figure 3.**
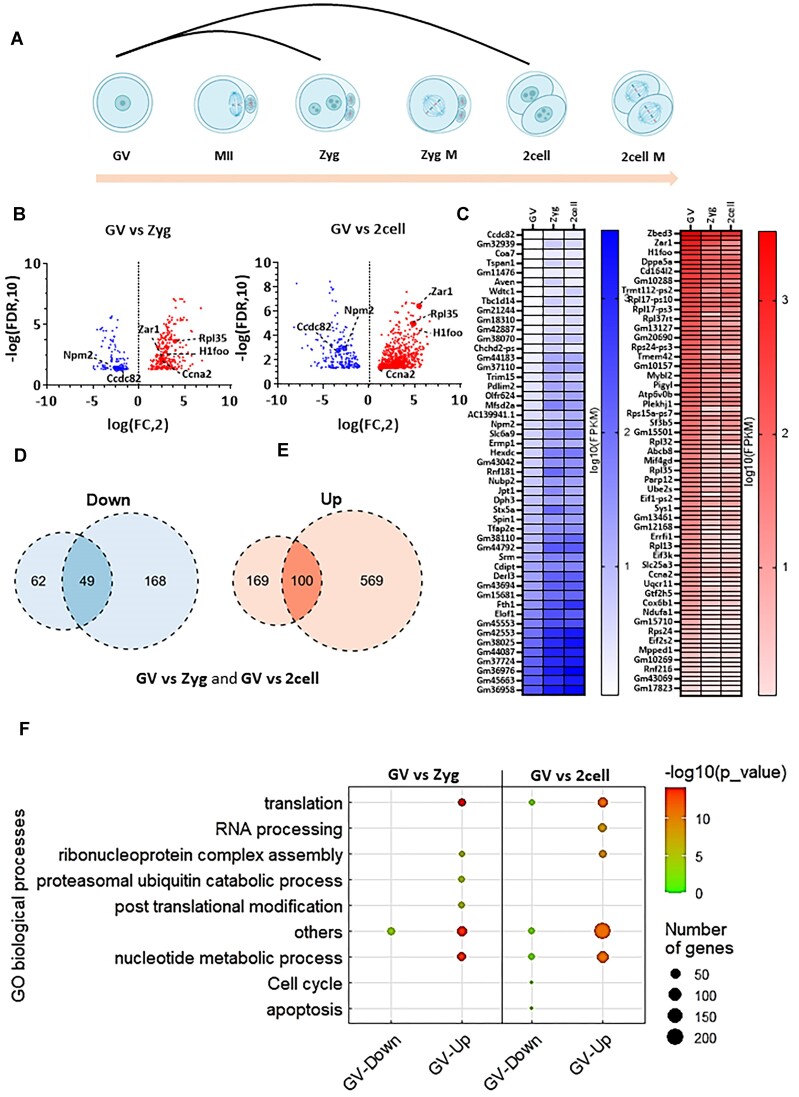
Polysome occupancy is higher in the oocyte interphase compared to embryo interphase. (**A**) Scheme of comparison of meiotic oocyte interphase with embryonic interphase. (**B**) Differential mRNA translation analysis of GV versus 1 and two-cell embryo stages. Volcano plots displaying candidate transcripts differentially enriched in polysomal fractions of oocytes and embryos from meiotic interphase and first mitotic interphases, highlighting those with FC > 2 (red) and FC < 2 (blue), adjusted *P*< 0.05. Dashed lines indicate candidate mRNAs translated in interphases compared. Connected to Supplementary File 4. (**C**) Heatmaps of Subset of mRNAs down and up regulated in oocyte interphase compared to embryo interphase. Connected to Supplementary File 4. (**D**) Venn diagram showing the number downregulated genes in oocyte interphase compared to embryo interphases. Connected to Supplementary File 4. (**E**) Venn diagram showing the number of upregulated genes in oocyte interphase compared to embryo interphases. Connected to Supplementary File 4. (**F**) Dot plot of top differentially translated gene transcripts and gene ontology (GO) analysis from (B). by WebGestalt for each cluster according to the top ranked genes for each cluster. The sizes and colours of the dots represent the number of genes and –log_10_-transformed *P*-values respectively. Connected to Supplementary File 5.

Our comparative analyses of GV oocytes with early embryos in interphase (Figure 3A) consistently showed (with respect to Figure 1B–E) that the number of actively translating mRNAs in GV oocytes was significantly higher than in the zygote, with the highest difference being observed in the comparison with the two-cell stage (569 genes) (Figure 3B–E and Supplementary File 4). Translation of 49 and 100 genes were constitutively down- and upregulated, respectively, in zygotes compared to GV oocytes (Figure 3B–E and Supplementary File 4). GO analysis showed that the translation of genes involved in translational processes was significantly higher in GV oocytes when compared to the zygote and two-cell stages (Figure 3F and Supplementary File 5).

**Figure 4.**
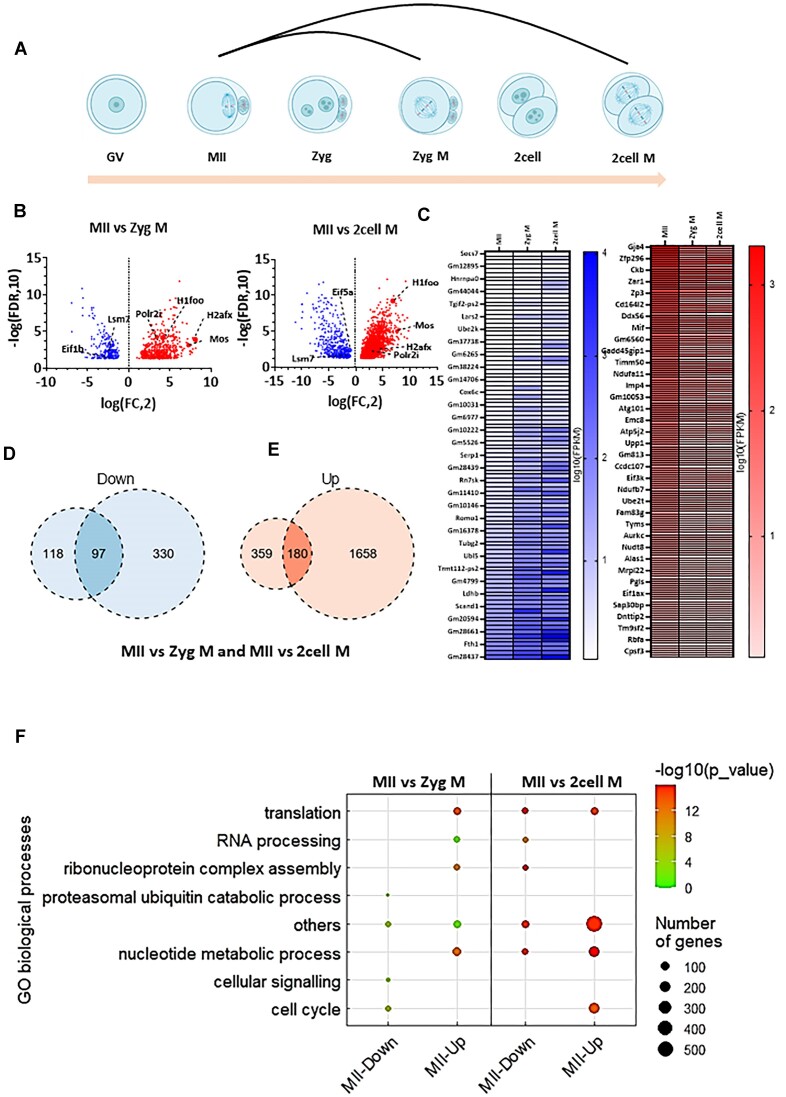
Meiotic M-phase has significantly higher translational activity than mitotic M-phases. (**A**) Scheme of comparison of meiotic M-phase with embryonic mitoses. (**B**) Differential mRNA translation analysis of meiotic M-phase versus first and second mitotic M-phases. Volcano plots displaying candidate transcripts differentially enriched in polysomal fractions of oocytes and embryos from interphase and M-phase comparisons, highlighting those with FC > 2 (red) and FC < 2 (blue), adjusted *P*< 0.05. Dashed lines indicate candidate mRNAs translated in M-phases compared. Connected to Supplementary File 6. (**C**) Candidate mRNAs commonly downregulated and upregulated in M-phases. Connected to Supplementary File 6. (**D**) Venn diagram showing the number of downregulated genes in MII phase compared to first mitotic M-phase. Connected to Supplementary File 6. (**E**) Venn diagram showing the number upregulated genes in MII phase compared to second mitotic M-phase. Connected to Supplementary File 6. (**F**) Dot plot of top differentially translated gene transcripts and gene ontology (GO) analysis from (B). by WebGestalt for each cluster according to the top ranked genes for each cluster. The sizes and colours of the dots represent the number of genes and –log_10_-transformed *P*-values, respectively. Connected to Supplementary File 7.

When we compared active translation between meiotic metaphase MII and mitotic metaphases (Figure 4A), as expected from Figure 1B, the number of actively translating mRNAs in meiosis was significantly higher than first and second embryonic mitoses (Figure 4B, C). Out of 1838 identified genes in two-cell M and 539 genes in zygote M, only 180 genes were constitutively upregulated; similarly, out of 427 genes in two-cell M and 215 genes in zygote M, only 97 were constitutively downregulated when compared to meiosis (Figure 4C–E and Supplementary File 6). GO analysis showed that the translation of genes involved in translational processes were significantly higher in the MII stage (Figure 4F and Supplementary File 7).

**Figure 5.**
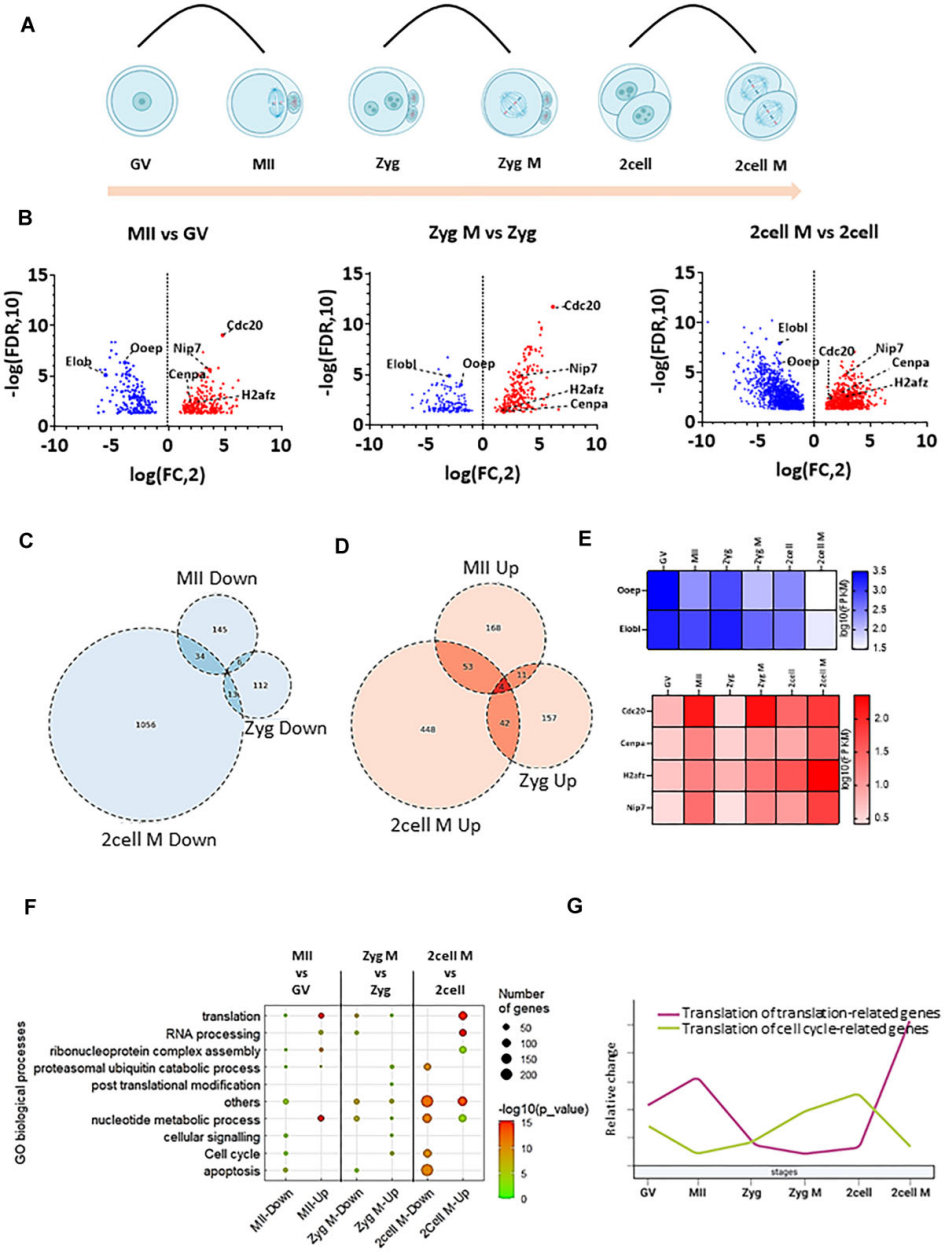
Translational regulation is significantly enriched in meiosis and 2nd embryonic mitosis. (**A**) Scheme of interphases and M-phases comparisons. (**B**) Differential gene expression analysis of M-phase versus interphase of oocytes and embryos. Volcano plots displaying candidate transcripts differentially enriched in polysomal fractions of oocytes and embryos from interphase and M-phase comparisons, highlighting those with FC > 2 (red) and FC < 2 (blue), adjusted *P*< 0.05. Dashed lines indicate candidate mRNAs translated in M-phases compared. See also Supplementary Figure 4 for candidate mRNA validation. Connected to Supplementary File 8. (**C**) Venn diagram showing the number of downregulated genes in M-phase compared to interphase. Connected to Supplementary File 8. (**D**) Venn diagram showing the number upregulated genes in M-phase compared to interphase. Connected to Supplementary File 8. (**E**) Candidate mRNAs commonly downregulated and upregulated in M-phases. Connected to Supplementary File 8. (**F**) Dot plot of top differentially translated gene transcripts and gene ontology (GO) analysis from (B). by WebGestalt for each group according to the top ranked genes for each cluster. The sizes and colours of the dots represent the number of genes and –log_10_-transformed *P*-values, respectively. Connected to Supplementary File S9. (**G**) Line graph derived from the dot plot (Figure 3F) highlighting the translation of cell cycle and translational gene in each group. Connected to Supplementary File 5.

We then asked how mRNA translation behaves in M-phases in connection to relevant interphases of the oocyte and embryo. To answer this question, we compared the polysome bound mRNA of M-phase with its corresponding interphase stage (Figure 5A). The highest difference among translatomes of M-phase and corresponding interphases was found between two-cell M and two-cell (Figure 5B–D and Supplementary File 8). Next, we analysed if specific genes were uniformly translated in the interphases or M-phases, however, we found that only *Cdc20, CenpA, H2afz* and *Nip7* mRNAs were constitutively translated and *Ooep, Elob1* mRNAs suppressed in translation in the M-phase (Figure 5B). Gene ontology analysis showed that translation of mRNAs coding for proteins involved in protein synthesis were highly enriched in M-phases of meiosis and the second mitosis (Figure 5F, G and Supplementary File 8&9). Conversely, the translation of genes involved in the cell cycle regulation were highest in the GV and two-cell interphases (Figure 5F, G and Supplementary File 8&9).

Collectively, our analysis indicates that translation of maternally stored mRNAs is significantly higher in GV and MII stage compared to the zygote and two-cell stages. Additionally, we show that translation of the subset of mRNAs is highly dynamic, stage specific and higher in oocytes than in early embryos. The most significant translatome dynamic occurs in meiosis and the second embryonic mitosis. Our data clearly indicate that the translation of maternal mRNAs is temporally regulated in connection to the cell cycle and developmental stage.

### Increased activity of eEF2, 4E-BP1 and mTOR translational pathways during M-phase

**Figure 6.**
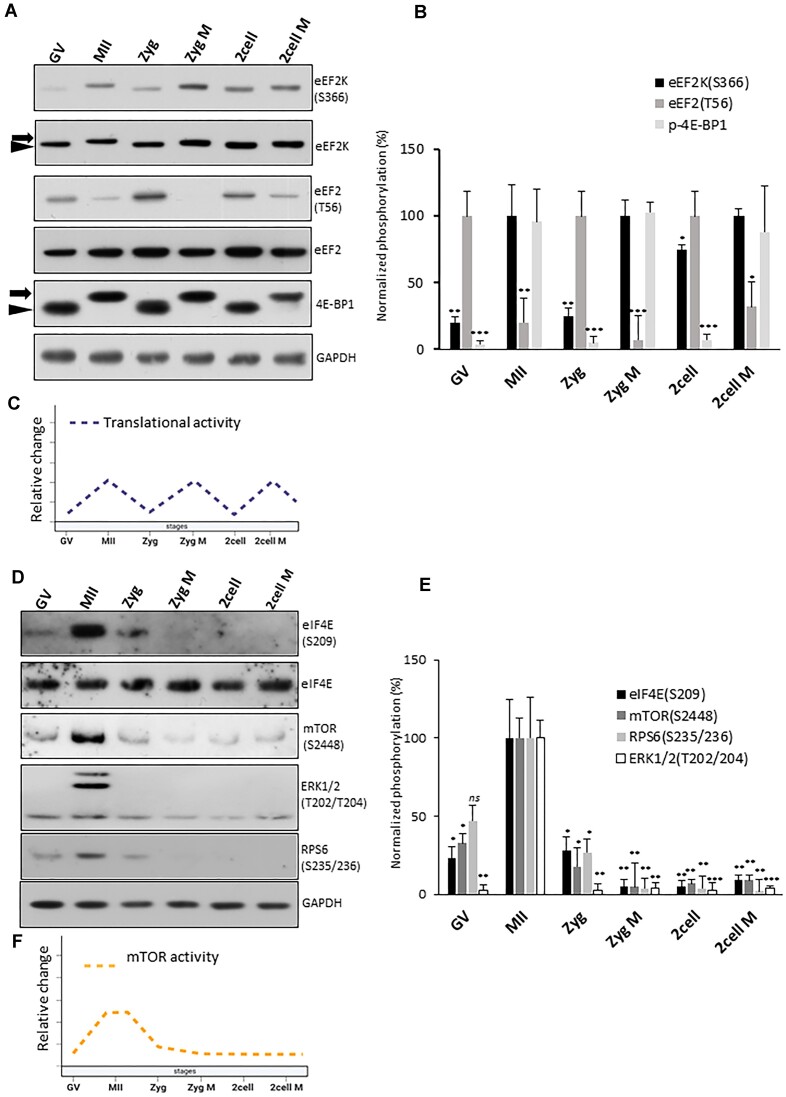
Increased activity of eEF2, 4E-BP1 and mTOR translational pathways during M-phase. (**A**) Immunoblot analyses of the key protein for cap-dependent translation show activity in M-phase. Arrow denotes phosphorylated and arrowhead for total form of protein. (**B**) Normalized densitometric values from components from (A). Data are represented as the mean ± s.d.; values obtained for relevant. stage with highest intensity was set as 100%. Data are represented as mean ± s.d.; *P< 0.05; **P< 0.01; ***P< 0.001 according to Student's *t*-test; from three biological replicates. (**C**) Scheme representing the active translation derived from the (A) and (B). (**D**) Western blot analysis of the key proteins for mTOR-related pathways. (**E**) Normalised densitometric values of immunoblot of (D). Data are represented as mean ± s.d.; MII set as 100%; *P< 0.05; **P< 0.01; ***P< 0.001 according to Student's *t*-test; from three biological replicates. (**F**) Scheme representing the mTOR activity derived from the (D) and (E).

The phosphorylation of 4E-BP1 by mTOR results in the release of eIF4E, enabling its interaction with eIF4G and the formation of the eIF4F complex, thereby facilitating cap-dependent translation initiation. To examine the relationship between mTOR signaling and translation during oocyte and embryo development, we conducted immunoblotting (IB) to assess the status of key translational regulators, including 4E-BP1, eukaryotic elongation factor kinase (eEF2K), and its downstream substrate, elongation factor eEF2. We showed that 4E-BP1 was uniformly hyper-phosphorylated during the M-phases, independent of developmental stage, leading to its inability to suppress initiation eIF4F complex formation. (Figure 6A–C). Additionally, the translational and elongation axis consistently exhibited higher activity during the M-phases across all developmental stages. (Figure 6A–C). Our current data also showed that specific mRNAs were actively translated during the M-phase (Figure 5B). Further IB analysis of the additional translational regulators mTOR, RPS6, ERK and translation initiation factors (14,26) showed the highest activity in the MII oocyte (Figure 6D–F and Supplementary Figure 5), correlating with a higher number of mRNAs being translated during this stage (Figure 4). Similarly, we observed that mRNA coding for components of the mTOR pathway (AKT, RPS6) and a number of eukaryotic initiation factors, abundantly occupied polysomes in MII oocytes, with a decreasing trend towards the two-cell M embryo stage (Supplementary Figure 5). To our knowledge, these results provide the first evidence of a uniform activation pattern of the key translation initiation and elongation factors linked to early developmental and cell cycle stages. Furthermore, the obtained data indicate variability of translation and activity of the key translational factors throughout meiotic maturation and early embryo development.

### Modulation of eEF2K/ eEF2 axis negatively influences embryo development

**Figure 7.**
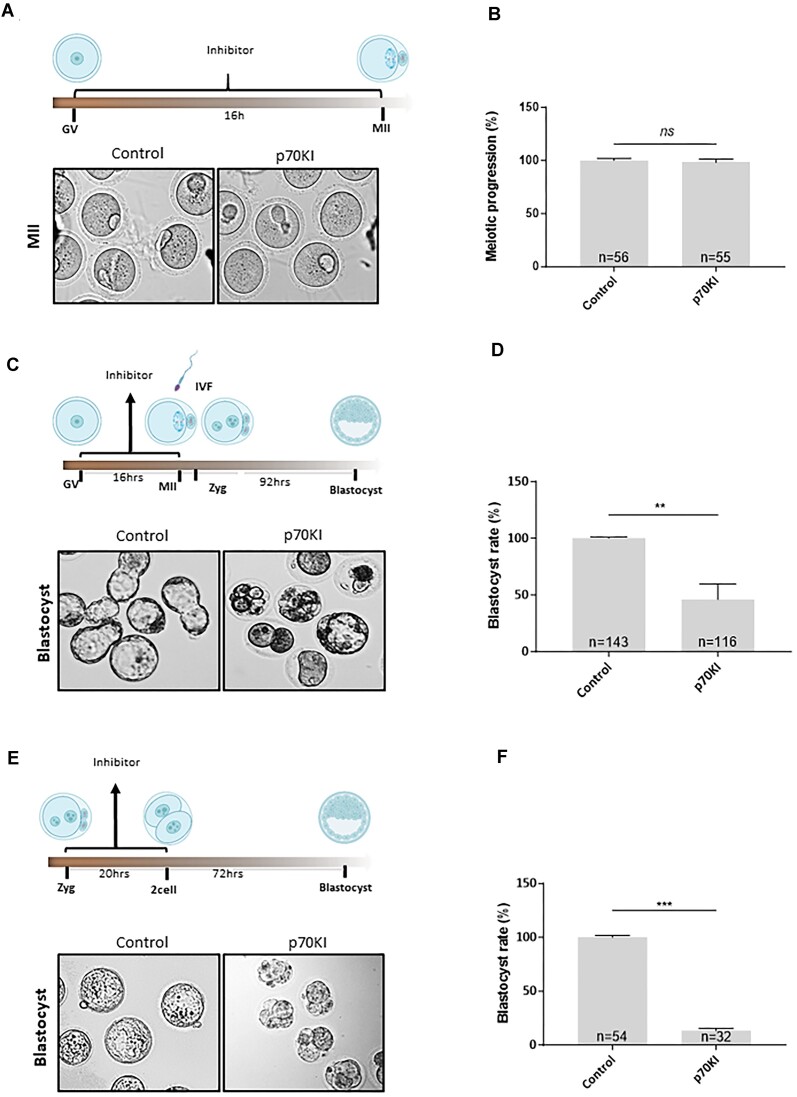
Modulation of eEF2K/ eEF2 axis negatively influences embryo development. (**A**) Scheme for inhibitor treatment of oocytes and impact on meiotic maturation. Representative images of meiotic progression of oocytes treated by 5μM p70KI inhibitor during meiotic maturation. For effect of inhibitor on the eEF2 phosphorylation see Supplementary Figure 6A, B. (**B**) Quantification of oocyte progression from GV to MII stage after inhibitor treatment. Data represented as mean ± s.d.; Student's *t*-test: *ns*, nonsignificant; from three biological replicates with presented *n*. (**C**) Scheme of inhibitor treatment in oocytes and followed by IVF. Representative image of embryo development after inhibitor treatment (p70KI) and IVF; *n* ≥ 3. (**D**) Quantification of blastocyst formation after inhibition of eEF2 during oocyte progression followed by IVF. Data are represented as mean ± s.d.; **P< 0.01 according to Student's *t*-test; from three biological replicates with presented *n*. For evaluation of fertilization see Supplementary Figure 6C. (**E**) Scheme for inhibitor treatment in embryos. Representative image of embryo development after 5 μM inhibitor treatment (p70KI); *n* ≥ 3. (**F**) Quantification of blastocyst formation after inhibition of eEF2 at zygote. Data are represented as mean ± s.d.; **P< 0.01 according to Student's *t*-test; from three biological replicates with presented *n*. For evaluation of two-cell development, see Supplementary Figure 6D. For additional inhibitor treatment see Supplementary Figure 7.

Based on the results indicating the activation of eEF2 in oocytes and embryos during M-phase (Figure 6A–C), we investigated whether inactivation of eEF2 would affect the meiotic or developmental competence of oocytes and embryos, respectively. To achieve eEF2 inhibition, we employed continuous activation of eEF2K via inhibition of S6K1 using a selective p70 ribosomal S6 kinase (S6K1) inhibitor (p70KI) and eEF2 inhibitor, ETA compound, (27,28); leading to increased eIF2 phosphorylation at Ser56 (Supplementary Figure 6A, B and Supplementary Figure 7A, B). Thus, resulting in eEF2 inhibition (29). Our findings revealed no discernible inhibitor effect on meiotic progression nor fertilization (Figure 7A, B and Supplementary Figure 6C and Supplementary Figure 7C, D, F). However, the induced change in eEF2 activity negatively impacted the ability of *in vitro* fertilized oocytes to accomplish preimplantation embryo development to the blastocyst stage (Figure 7C, D). Additionally, we treated zygotes with p70KI and analyzed developmental competence up to the two-cell stage. We found no visible effect of eEF2 inhibition on development to the two-cell stage (Supplementary Figure 6D and Supplementary Figure 7E, G), however, blastocyst development rates were decreased significantly (Figure 7E, F and Supplementary Figure 7E, G).

Collectively, our data clearly suggest that altering the activity of translation elongation during the earliest stages of development (during oocyte meiotic maturation and during the oocyte-embryo transition) has a detrimental effect on the preimplantation developmental potential of oocytes and zygotes.

## Discussion

In this study, we used a genome-wide approach to identify cell cycle-dependent translation of maternal mRNAs. In the absence of transcription, translation is the major regulator of oocyte and early embryonic development (7,30,31). Our results reveal that global translation varies throughout the cell cycle, specifically during the studied interphases and M-phases, independently of oocyte meiosis or embryonic mitoses. Although translation is globally decreased in M-phases, our analysis of M-phase translatomes reveal surprising uniformity in the activation of translation initiation and elongation players, that promote the translation of a subclass of maternal mRNAs. Throughout the cell cycle, it is critical that certain proteins are synthesized rapidly and in sufficient quantities to ensure an intact continuum of developmental progression (32). Our findings support the importance of translational regulation in this process and suggest that specific subsets of maternal mRNAs are selectively translated during M-phases.

Our study also reveals that certain subsets of mRNAs are part of highly dynamic translational clusters, with the translational rate of 12 observed clusters changing significantly during development and promoting the synthesis of specific proteins essential for the current or subsequent stage. Interestingly, subclasses of mRNAs belonging to different biological processes were expressed in temporally coordinated patterns, exhibiting a few dominant biological processes at specific stages. For example, translation of mRNAs encoding translation factors were most active in the MII oocyte and the two-cell M embryo, positively correlating with increased translation of specific mRNAs associated with the completion of meiosis and the maternal-zygotic transition (MZT) (7). Interestingly, translation of mRNAs encoding cell cycle regulatory factors decreased from the GV oocyte to the MII oocyte and peaked in their translational activity at the two-cell stage, with minimal translation in the MII oocyte or two-cell M embryo. To our surprise, we found only six uniformly translating mRNAs in the M-phases, indicating specific translation in each metaphase. We also observed that a large number of mRNAs were not commonly translated in the M-phases, as in interphases, indicating differential contribution to the meiotic and the first two mitotic M-phases. However, mRNAs encoding essential cell division factors such as CDC20, CENP A, and H2AFZ (33–35), were significantly translated in all three M-phases examined, as were the downregulated oocyte-specific transcripts OOEP and ELOBL (36). Importantly, GO profiles associated with apoptosis showed a decreasing trend, indicating no negative effects of *in vitro* manipulation.

The observed increase in the number of gene transcripts at the two-cell stage could be due to onset of the major genome activation. In mice, the maternal-zygotic transition (MZT) occurs at the late two-cell stage, where developmental control is transferred to the zygotic genome (reviewed in (37). This is accompanied by a significant increase in mRNAs encoding factors involved in translation processes. Interestingly, translation of protein synthesis associated factors are significantly reprogrammed in the MII oocyte and post-two-cell stage embryo. Our study also revealed that the most robust translational changes were detected in the second mitotic M-phase, suggesting that transcriptional reprogramming in the second embryonic interphase is reflected by accompanying and related translational changes. This finding is consistent with observations in the bovine model, where MZT occurs at the eight-cell stage (38). Consistently, the post two-cell stage embryo is significantly affected by the downregulation of the eIF2K/eIEF2 signalling axis. Taken together, our results provide new insights into the dynamic regulation of translation during oocyte and early embryonic development, highlighting the importance of translational control in ensuring the timely and germane progression of the early developmental stages.

Additionally, the study showed significant quantitative changes in the translatomes during oocyte and embryonic development with uniform oscillations observed in the activity of the translational machinery components, including translation initiation and elongation factors. This suggests that the protein synthesis machinery may adapt to optimize the translation of specific mRNAs. Similarly, Smith and Proud, 2008 have reported low phosphorylation status of eEF2 by the inactivation of eEF2K due to elevated calcium levels (40), controlled by the cyclin-dependent kinase (CDK1) in mitotic cells (39). This is consistent with our finding that eEF2 is dephosphorylated and active during the M-phases of meiosis and mitosis, while the mTOR-eIF4F translation pathway is highly activated during NEBD (14). Such activation disappears post-fertilization and reactivates in the 8- to 16-cell stage mouse embryo (41). Moreover, the dominant activity of mTOR1, RPS6, eIF4E, and ERK1/2 occurs in the oocyte, which correlates with the highest observed levels of global translation. It has been shown that mTOR1 and RPS6 phosphorylation play a role in translational control of a subclass of mRNAs containing the 5′-tract oligopyrimidine sequence (5′ TOP) and this level of regulation may confer greater specificity to the ribosome (12,14,42,43). Similarly, ERK1/2 triggers meiosis-dependent mRNA translation. In addition to the uniform mode of increased translational initiation/elongation activity in M-phases, the mTOR1 signaling pathway is unique to oocyte development, which could distinguish meiotic from mitotic translation and represents cell type-specific translation.

Inhibition of the mTOR pathway differentially affects polysomal recruitment of newly translated mRNAs, which are either mTOR-dependent (44) or mTOR-independent (21,45–49). Similarly, ERK1/2 triggers meiosis-dependent mRNA translation (26). Our results highlight the possibility of translation being reprogrammed to promote translation in a cell type- and cell cycle-dependent manner. This may explain the observed discrepancy between the decrease in global translation and the activation of translation initiation and elongation in meiotic and mitotic M-phases.

In summary, our work sheds light on mRNA translation encoding components of metabolic pathways whose periodic expression has not been previously demonstrated. It is tempting to speculate that the discovered mRNAs and encoded proteins play, as yet, unknown roles in the progression or regulation of the mammalian cell cycle and early development. Along with the observation that temporal patterns are present at the level of translation and oscillatory activity of specific translational players simultaneously ensures the transition of different cell types, cell cycles, and developmental stages.

## Supplementary Material

gkad996_Supplemental_Files

## Data Availability

The data underlying this article are available the Gene Expression Omnibus at https://www.ncbi.nlm.nih.gov/geo/ under the accession number GSE230016.
