# Supplementary material for: The translational oscillation in oocyte and early embryo development
Source: Nucleic Acids Res. 2023 Nov 10;51(22):12076–91. doi: 10.1093/nar/gkad996 (PMC10711566; doi:10.1093/nar/gkad996)
Supplement: gkad996_Supplemental_Files [file gkad996_supplemental_files.zip › Supplementary Legends and Figures R1.pdf]

SUPPLEMENTARY FIGURES LEGENDS

Supplementary Figure 1. Analysis of translational stress in the oocytes and early embryos used in the study.
A. DAPI stained oocytes and early embryos in the interphase and M-phase. Scale bar, 15 μm. The dashed lines represent cell cortex. The lower row shows the zoomed nuclei/chromosomal area.
B. Analysis of activity of the translational stress marker eIF2a (S51) by immunoblotting. GAPDH was used as a loading control. Representative image from three biological replicates.
C. Ratio of the eIF2a activity in the oocyte and early embryo development. Data are represented as the mean ± s.d.; ***p*<0.01; *ns*, nonsignificant; Student's *t*-tests; n≥5.
D. A comparative analysis of the impact of Nocodazole (Noco) on the activity of 4E-BP1, eIF2a, and eEF2K in naturally progressing oocytes. GAPDH was used as a loading control. Representative image from three biological replicates.
E. Immunoblotting analysis of effect of stressor $NaAsO_2$ on the phosphorylation of eIF2a, eEF2 and 4E-BP1 in the embryonic M-phases. GAPDH was used as a loading control. Representative image from three biological replicates.

Supplementary Figure 2. Validation of ribosomal fractionation for presence of 18S and 28S rRNA.
A. qRT-PCR analysis of distribution of 18S rRNA in non-polysomal and polysomal fractions in different developmental stages of oocytes and embryos. Data are represented as the mean ± s.d.
B. qRT-PCR analysis of distribution of 28S rRNA in non-polysomal and polysomal fractions in different developmental stages of oocytes and embryos. Data are represented as the mean ± s.d.

Supplementary Figure 3. RNAseq analysis of ribosomal fractionation from oocytes and early embryos.
A. Principal component analysis of polysomal and non-polysomal RNA in different stages of oocyte and early embryo development.
B. Total number of polysome occupied RNAs >0.1 FPKM in the oocytes and early embryos. No significant change in the total number of gene expressed in each stage. However, genes are differentially expressed based on the cell cycle stages.
C. Heat map showing the differential gene expression of GV and MII stage.
D. Heat map showing the differential gene expression of Zygote and Zygote M stage.
E. Heat map showing the differential gene expression of 2cell and 2cell M stage.

Supplementary Figure 4. Validation of datasets from RNA-seq polysomal fractions.
Analysis of *Cdc20, Ooep, Rpl35, Mos and Polr2i mRNA* expression in whole transcriptome in the different stages. Data are represented as mean ± s.d.; ns-nonsignificant; *p<0.05 according to Student's *t*-test; from three biological replicates. Polysome bound *Ccdc20, Ooep, Rpl35, Mos and Polr2i mRNA* detected by RNA-sequencing. Data are represented as mean ± s.d.; ***p<0.001 according to Student's *t*-test; from four biological replicates. qRT-RT analysis of *Cdc20, Ooep, Rpl35, Mos and Polr2i* mRNA presence in the polysomal fractions. Data are represented as mean ± s.d.; ****p*<0.001 according to Student's *t*-test; from three biological replicates. Immunoblot analysis of CDC20, RPL35, MOS, and POLR2I protein expression in the different developmental stages. Representative image from at least two independent replicates.

Supplementary figure 5. The mTOR1 translational pathway is abundant in the oocyte and decreases in early embryonic development.
A. Polysomal occupation of mRNAs coding for canonical mTOR1 translational pathway. Data are represented as the mean ± s.d.
B. Polysomal occupation of mRNAs coding for eukaryotic initiation factors. Data are represented as the mean ± s.d.

Supplementary Figure 6. Validation of the eEF2 activity with p70KI.
A. Immunoblot analysis of phosphorylation status of eEF2 on Thr56 in the oocytes treated with vehicle (control) and p70KI in different concentration. Representative images from three biological replicates.
B. Normalized densitometric values of eEF2(T51) from components from A. Data are represented as the mean ± s.d.; values obtained for relevant. stage with lowest intensity was set as 100%. Data are represented as mean ± s.d.; *ns*, non-significant; ***p*<0.01 according to Student's *t*-test; from three biological replicates.

C. Quantification of fertilization rate after inhibition of eEF2 using 5 µM p70KI inhibitor during oocyte maturation followed by IVF.
Data are represented as mean ± s.d.; *ns*, nonsignificant according to Student's *t*-test; from three biological replicates with presented n.
D. Quantification of 2cell development after inhibition of eEF2 post fertilization. Data are represented as mean ± s.d.; *ns*, nonsignificant according to Student's *t*-test; from three biological replicates with presented n.

Supplementary Figure 7. Validation of the eEF2 activity with Exotoxin A (ETA).
A. Immunoblot analysis of phosphorylation status of eEF2 on Thr56 in the oocytes treated with vehicle (control) and ETA in different concentration, from three biological replicates.
B. Normalized densitometric values of eEF2(T56) from components from **A**. Data are represented as the mean ± s.d.; values obtained for relevant. stage with lowest intensity was set as 100%. Data are represented as mean ± s.d.; *p<0.05 according to Student's *t*-test; from three biological replicates.
C. Scheme of the experiment and representative images of MII stage oocytes after 16h of 72 nM inhibitor treatment.
D. Quantification of meiotic progression after inhibition of eEF2 during oocyte maturation. Data are represented as mean ± s.d.; *ns*, nonsignificant according to Student's *t*-test; from three biological replicates with presented n.
E. Scheme of the experiment and representative images of the blastocysts from 72 nM ETA treated oocytes.
F. Quantification of fertilization rate after inhibition of eEF2 during oocyte maturation followed by IVF. Data are represented as mean ± s.d.; *ns*, nonsignificant according to Student's *t*-test; from three biological replicates with presented n.
G. Quantification of blastocyst development after inhibition of eEF2 during oocyte maturation followed by IVF and then assessed further development. Data are represented as mean ± s.d.; *ns*, nonsignificant according to Student's *t*-test; from three biological replicates with presented n.

Supplementary Table 1: Supplementary tables of primers used for qPCR (**A**) and primary antibodies used in Immunoblotting, immunocytochemistry and PLA (**B**).

SUPPLEMENTARY FILE LEGENDS

Supplementary File 1. Mouse Polysome_normalized FPKM. A complete list of genes after normalization from RNAseq data are listed in this file. F1: Non polysome, F6: Polysome (highlighted as green). A, B and C represents the first, second and third replicates.

Supplementary File 2. (Fig. 2A) Clusters. Total genes and its average values used for the cluster classification.

Supplementary File 3. (Fig. 2B) GO Dot plot Cluster major. GO analysis and the combined group of GO terms along with "*p*" values of each cluster.

Supplementary File 4. (Fig. 3B, D, E) DE genes interphase vs interphase. List of up and down regulated genes, fold change, and "*p*" values compared to GV *vs*. zygote and GV vs 2cell stage.

Supplementary File 5. (Fig. 3 F) GO Dot plot Oocyte interphase *vs* Embryo interphase. GO analysis and the combined group of GO terms along with "*p*" values of up and down regulated genes compared to GV *vs*. zygote and GV *vs.* 2cell stage.

Supplementary File 6. (Fig. 4B C, D, E) DE genes meiosis *vs* mitosis. List of up and down regulated genes, fold change, and "*p*" values compared to MII vs zygote M and MII *vs*. 2cell M stage.

Supplementary File 7. (Fig. 4F) GO Dot plot Oocyte meiosis *vs* Embryo Mitosis. GO analysis and the combined group of GO terms along with "*p*" values of up and down regulated genes compared to MII *vs*. zygote M and MII *vs*. 2cell M stage.

Supplementary File 8. (Fig. 5B, C, D, E) DE genes M-phase *vs* Interphase. List of up and down regulated genes, fold change, and "*p*" values compared to MII vs GV, zygote M *vs*. Zygote and 2cell M *vs*. 2cell stage.

Supplementary File 9. File (Fig. 5F) GO Dot plot M-phase *vs* Interphase. GO analysis and the combined group of GO terms along with "*p*" values of up and down regulated genes compared to MII vs GV, zygote M vs. Zygote and 2cell M *vs*. 2cell stage.

Supplementary Figure 1

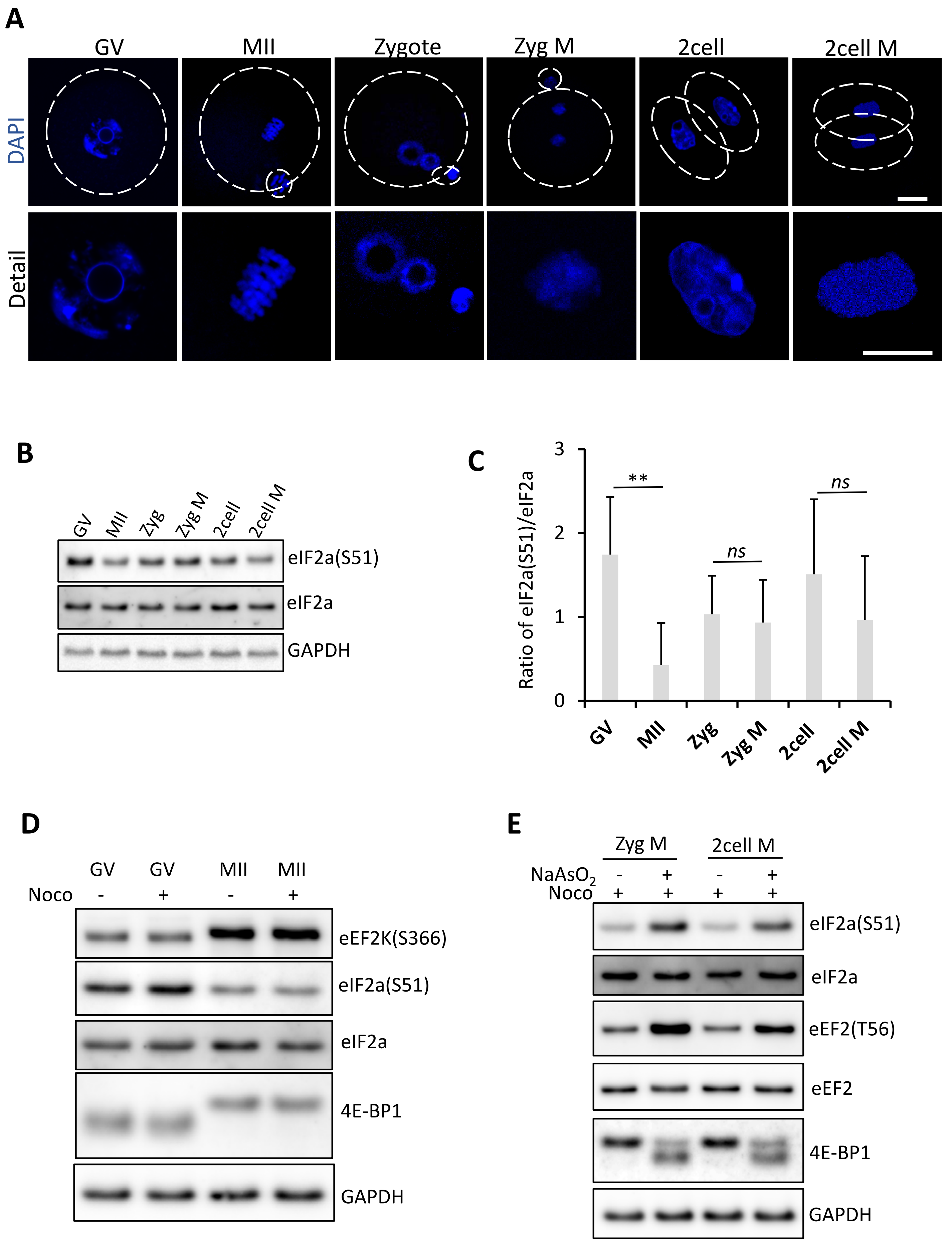

Supplementary Figure 2

A

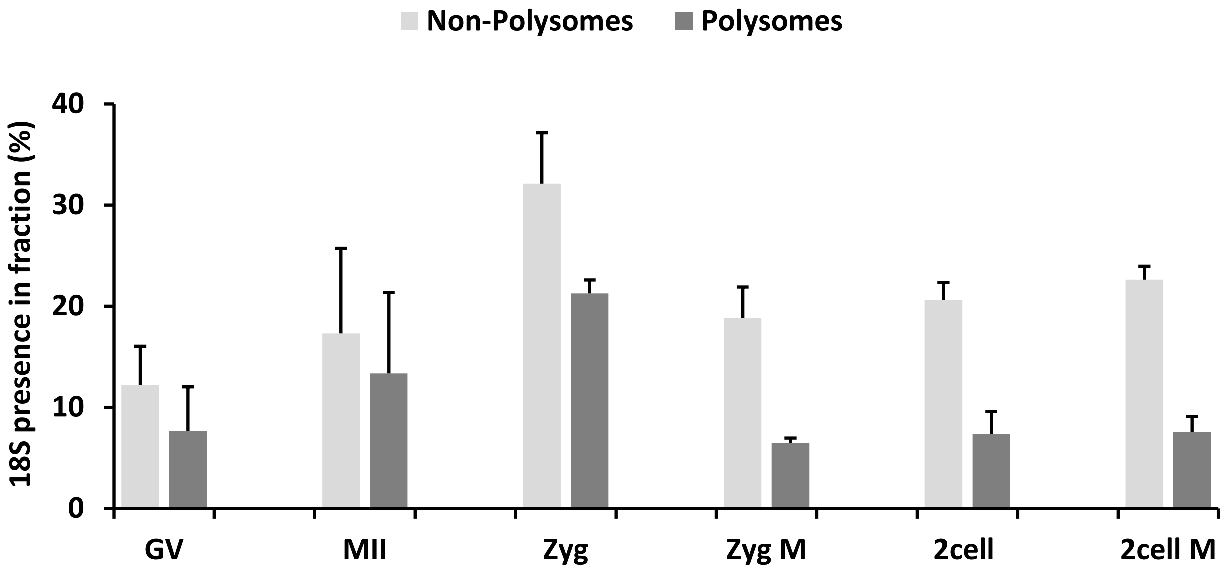

B

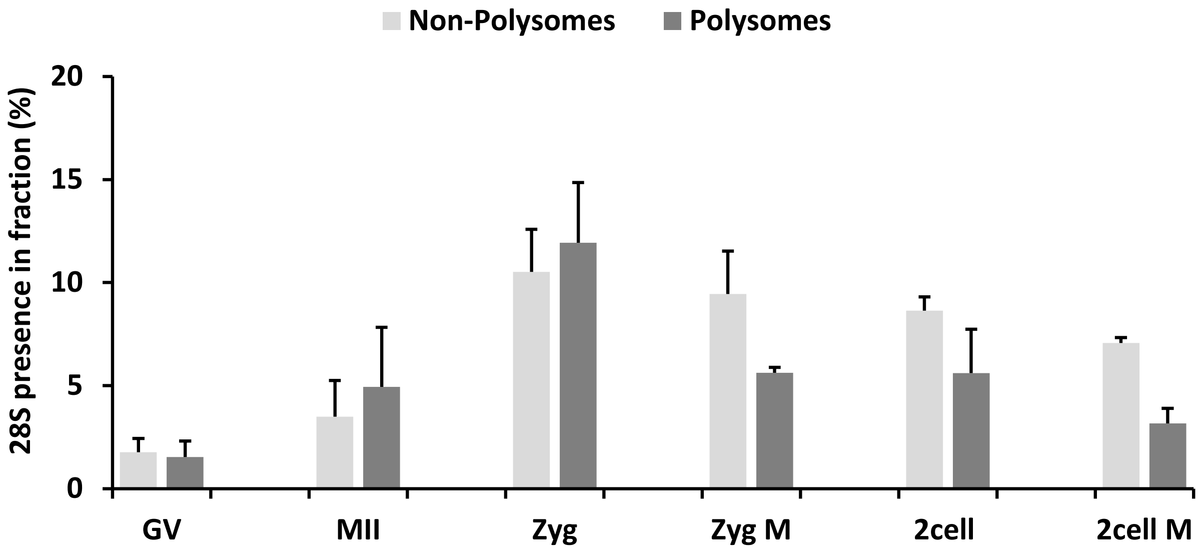

Supplementary Figure 3

A

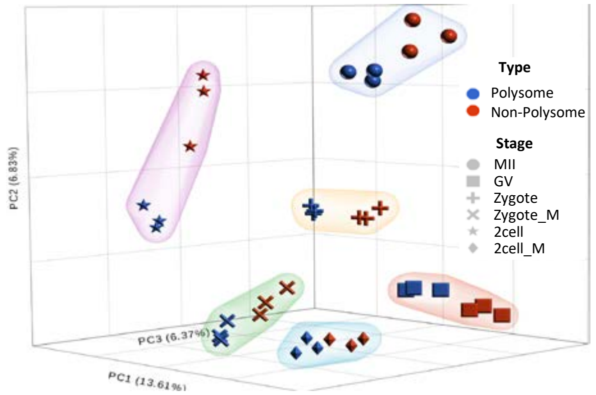

B

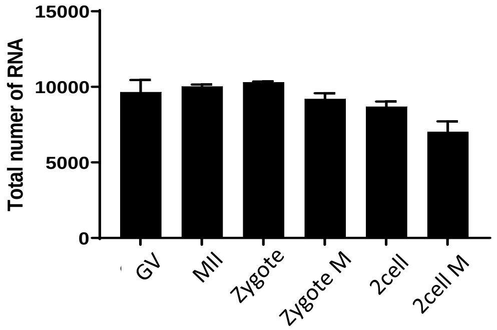

C

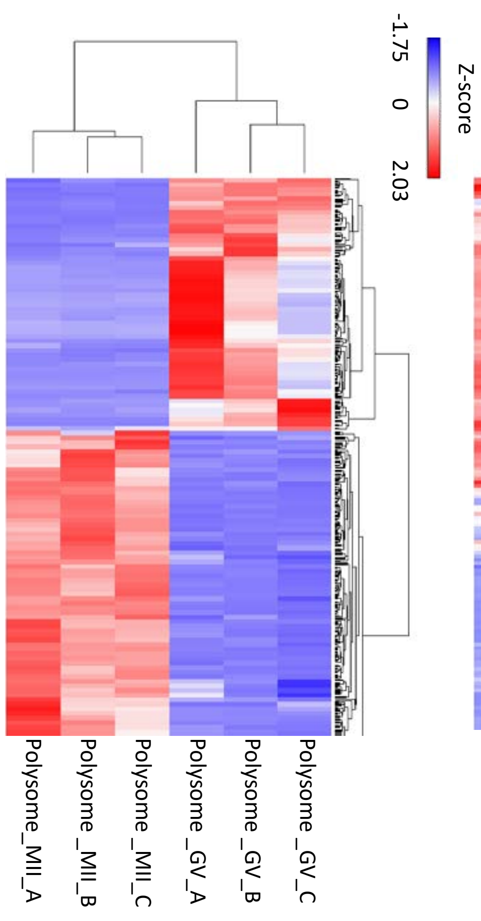

D

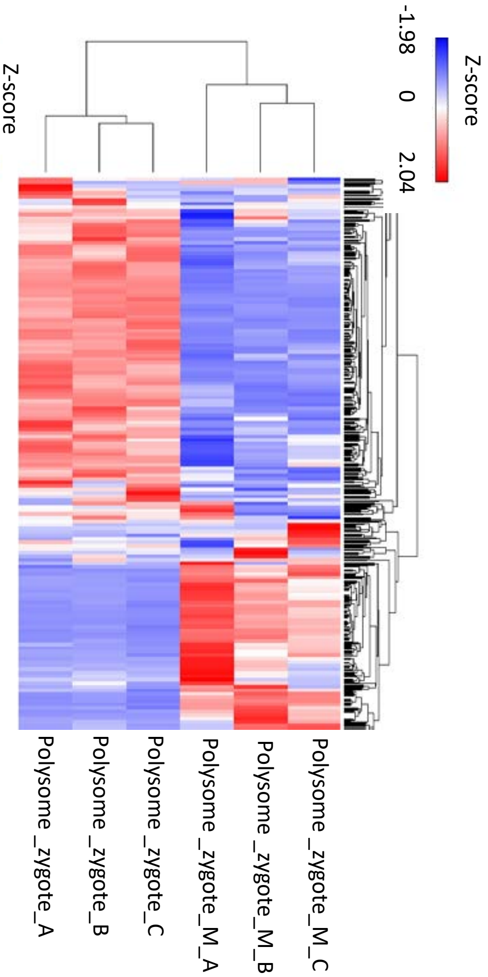

E

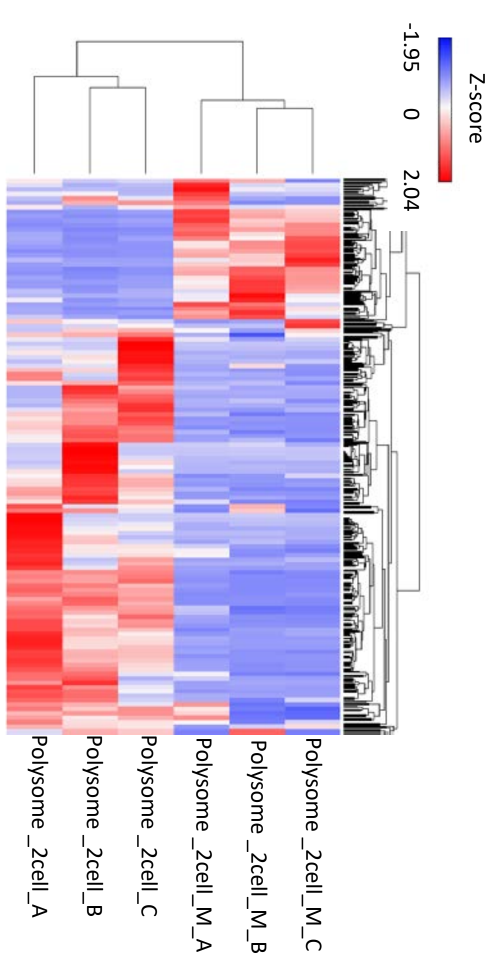

Supplementary Figure 4

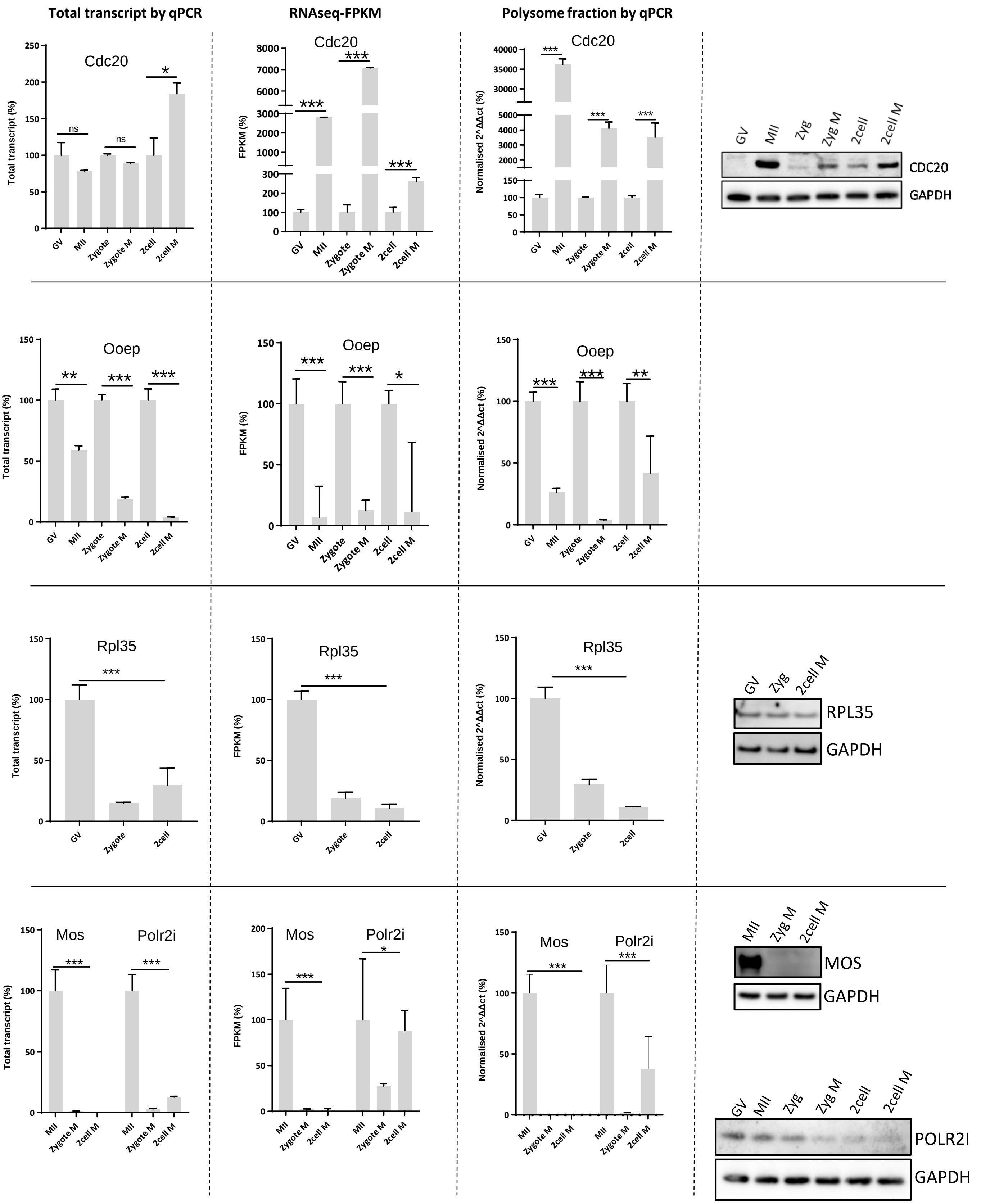

Supplementary Figure 5

A

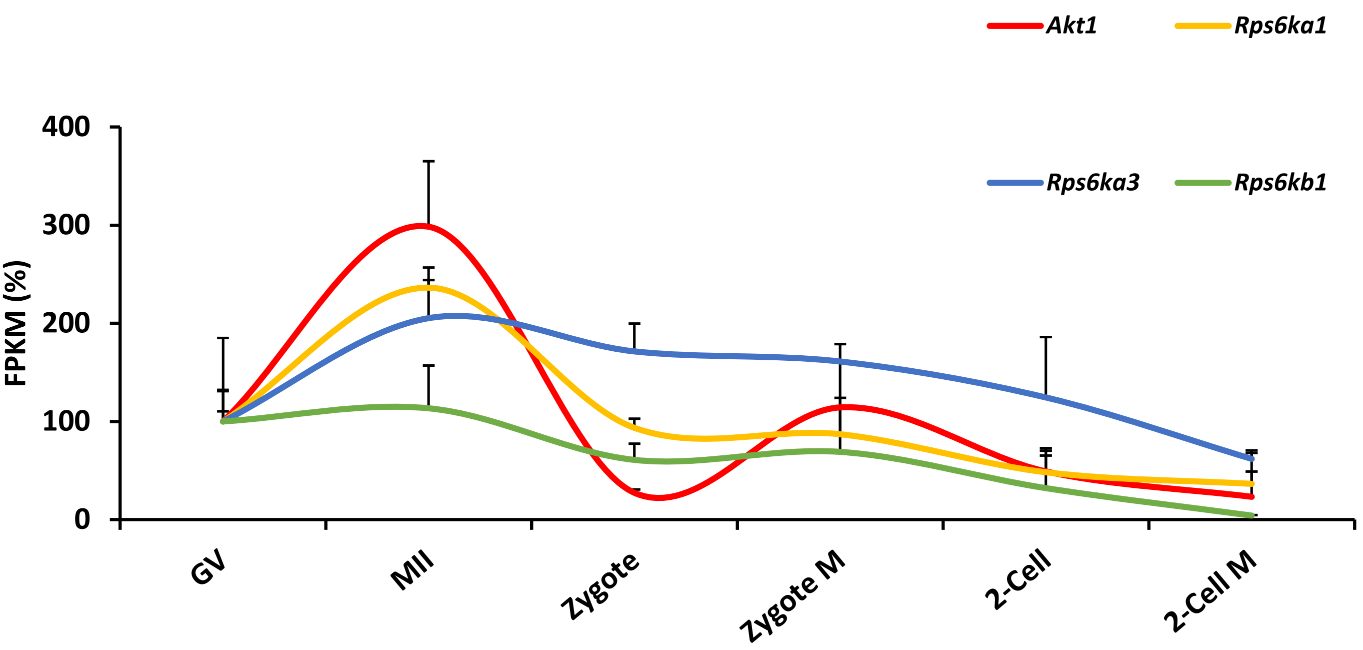

B

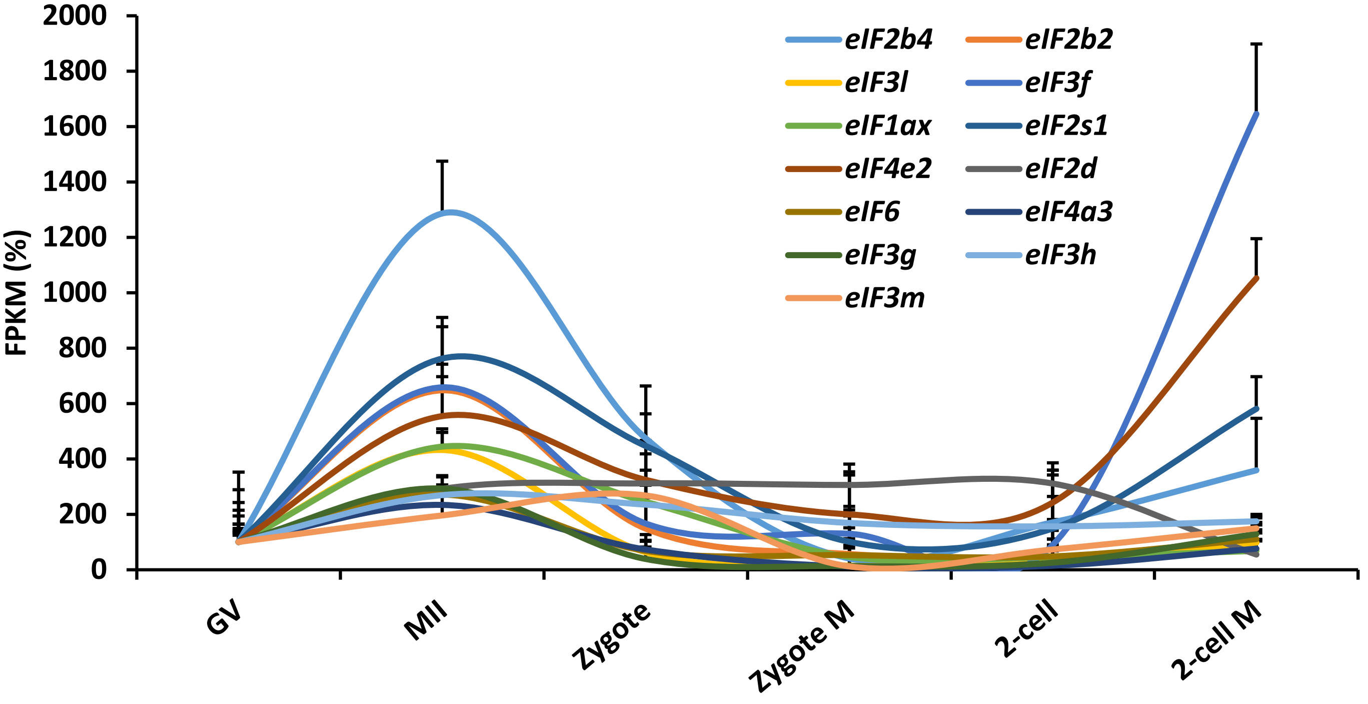

Supplementary Figure 6

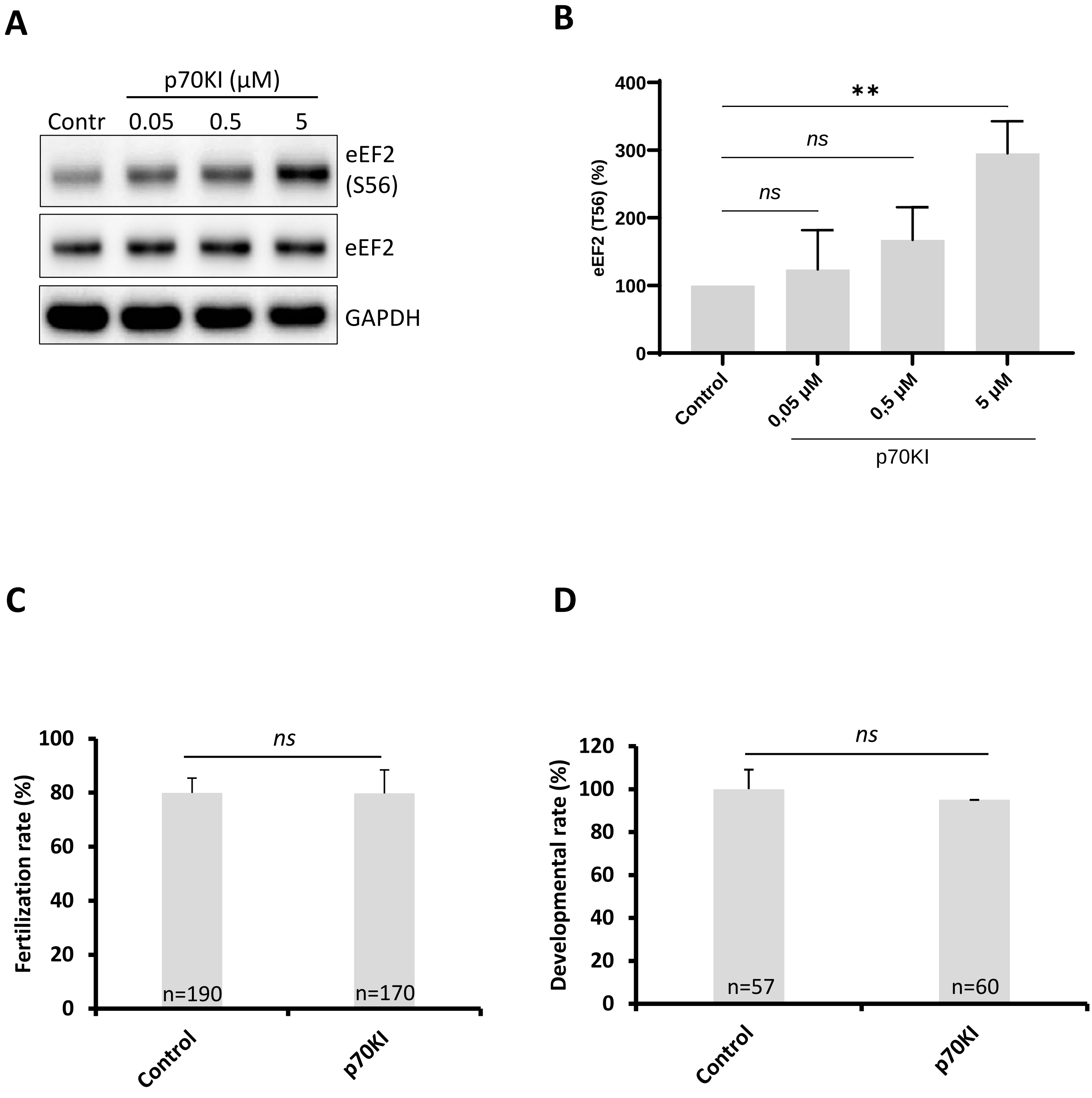

Supplementary Figure 7

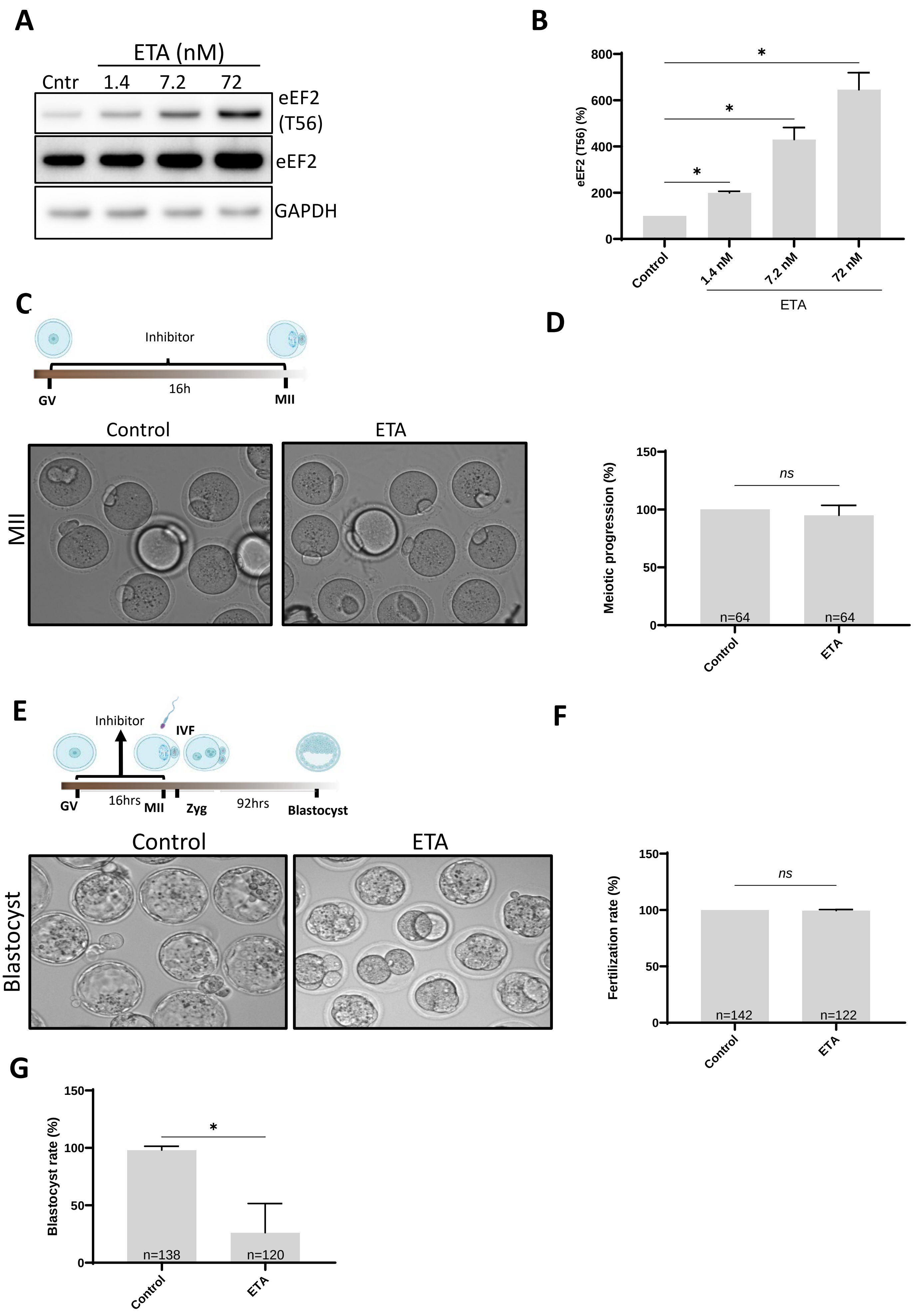

Supplementary Table 1

A

Official symbol (Gene)	Foward 5´-3´	Reverse 5´-3´	product size (bp)
Cdc20	GATCCTTGATGCCCCCGAAA	TGCAGGATGTCACCAGAACC	132
18S	CGCTCCACCAACTAAGAACG	CTCAACACGGGAAACCTCAC	110
28S	CTAAATACCGGCACGAGACC	TTCACGCCCTCTTGAACTCT	88
RPL35	GCCAAGATTAAGGCTCGGGA	GCGAACGACTCGTATCTTGGA	150
Mos	GTATAAAGCCACTTACCACGG	CAATGTTCAGTTCAGCCCA	107
Polr2i	CAGCCCAGTCGCTATGGAAC	CTGGGTCAGCTCGTCCACTT	214
Ooep	CTGTGTCCCTGAGACTTCGC	TGGTCTGTGCCTATGACCCT	115

B

Official symbol (Protein)	Cat. №	Manufacturer
eEF2K	3692	Cell Signalling
eEF2K(S366)	3691	Cell Signalling
eEF2	2332	Cell Signalling
eEF2(T56)	2331	Cell Signalling
4E-BP1	9644	Cell Signalling
eIF2a	9722	Cell Signalling
eIF2a(S51)	3398	Cell Signalling
mTOR (S2448)	5536	Cell Signalling
ERK (T202/T204)	9101	Cell Signalling
LMN A/C	SAB4200236	Sigma Aldrich
Histone H3 (Ser10)	9701	Cell Signalling
RPL24	PA562450	Thermo Fisher
RPS6	74459	Santa Cruz
RPS6 (S235/236)	4858	Cell Signalling
GAPDH	G9545	Sigma Aldrich
RPL35	SAB4500233	Sigma Aldrich
Mos	PA5-101081	Invitrogen
POLR2I	398049	Santa Cruz
